# Rapid Detection and Quantification of Plant Innate Immunity Response Using Raman Spectroscopy

**DOI:** 10.3389/fpls.2021.746586

**Published:** 2021-10-21

**Authors:** Pil Joong Chung, Gajendra P. Singh, Chung-Hao Huang, Sayuj Koyyappurath, Jun Sung Seo, Hui-Zhu Mao, Piyarut Diloknawarit, Rajeev J. Ram, Rajani Sarojam, Nam-Hai Chua

**Affiliations:** ^1^Temasek Life Science Laboratory, National University of Singapore, Singapore, Singapore; ^2^Disruptive and Sustainable Technologies for Agricultural Precision, Singapore-MIT Alliance for Research and Technology, Singapore, Singapore; ^3^Research Laboratory of Electronics, Massachusetts Institute of Technology, Cambridge, MA, United States

**Keywords:** Arabidopsis, carotenoids, elf18, flg22, pathogen-associated molecular pattern (PAMP), Raman spectroscopy, plant innate immunity (PTI)

## Abstract

We have developed a rapid Raman spectroscopy-based method for the detection and quantification of early innate immunity responses in Arabidopsis and Choy Sum plants. Arabidopsis plants challenged with flg22 and elf18 elicitors could be differentiated from mock-treated plants by their Raman spectral fingerprints. From the difference Raman spectrum and the value of *p* at each Raman shift, we derived the Elicitor Response Index (ERI) as a quantitative measure of the response whereby a higher ERI value indicates a more significant elicitor-induced immune response. Among various Raman spectral bands contributing toward the ERI value, the most significant changes were observed in those associated with carotenoids and proteins. To validate these results, we investigated several characterized Arabidopsis pattern-triggered immunity (PTI) mutants. Compared to wild type (WT), positive regulatory mutants had ERI values close to zero, whereas negative regulatory mutants at early time points had higher ERI values. Similar to elicitor treatments, we derived an analogous Infection Response Index (IRI) as a quantitative measure to detect the early PTI response in Arabidopsis and Choy Sum plants infected with bacterial pathogens. The Raman spectral bands contributing toward a high IRI value were largely identical to the ERI Raman spectral bands. Raman spectroscopy is a convenient tool for rapid screening for Arabidopsis PTI mutants and may be suitable for the noninvasive and early diagnosis of pathogen-infected crop plants.

## Introduction

Global food supply and security have been challenged by water scarcity and climate change, and the situation is further exacerbated by the projected increase in the world's population to about 10 billion by 2050 (Misra, 2014). The expected increase in food demand requires a corresponding increase in crop productivity and disruptive improvements in agricultural production systems. Crop productivity in the field is compromised by abiotic stresses as well as biotic stresses, and the potential agricultural yield losses caused by plant pathogens are about 16% globally (Oerke, 2006).

Several strategies have been implemented to mitigate the degradation of crop yield caused by plant diseases. The generation of pathogen-resistant plants by transgenic means (Dong and Ronald, 2019; van Esse et al., 2020) and the use of agrochemicals to confer disease resistance (Zhou and Wang, 2018) have met with limited success. In the field, rapid detection of pathogen-infected plants is an important first step in plant disease management. The early detection of infected plants would allow their rapid and selective removal, thus greatly reducing pathogen load and the opportunity for further disease spreading. A visual inspection of disease symptoms and the grading of their disease severity turn out to be a classical method of identifying infected plants. However, most disease symptoms usually manifest only at relatively later stages of infection when the pathogen load is already quite high. Therefore, over the last two decades, plant disease diagnosis has shifted from a visual inspection of symptom-based disease phenotype to molecular-based diagnostic procedures (Fang and Ramasamy, 2015; Martinelli et al., 2015). Nevertheless, these molecular methods suffer from several drawbacks such as a dependency on the availability of pathogen-specific gene sequences or antibodies. Their implementation requires time and is not amenable to on-site field application.

To obviate the drawbacks of molecular methods, several groups have explored the use of Raman spectroscopy for a rapid diagnosis of plant diseases (Yeturu et al., 2016; Egging et al., 2018; Farber and Kurouski, 2018; Farber et al., 2019, 2020a,b; Mandrile et al., 2019; Sanchez et al., 2020b). A Raman spectrum records the molecular vibrations of cellular metabolites without the use of a label or reagents. Differences in the Raman spectra of a diseased sample vs. that of the control sample are fingerprints, which reflect changes in cellular metabolites following pathogen infection. The acquisition of a Raman spectrum usually takes less than a minute and using a hand-held device, such a recording can be directly done on field crops (Gupta et al., 2020).

Raman spectroscopy-based detection and identification of bacterial, fungal, and viral diseases in plants have been reported (Yeturu et al., 2016; Farber and Kurouski, 2018; Farber et al., 2019; Mandrile et al., 2019; Sanchez et al., 2020b,c; Payne and Kurouski, 2021). Meanwhile, Raman fingerprints vary depending on the disease symptoms and the pathogens involved, in most cases, the Raman spectra collected from the diseased leaves primarily show changes in peak intensities corresponding to vibrational bands originating from carotenoids, pectin, phenylpropanoids, cellulose, and proteins. In most cases, irrespective of the responsible pathogen, a decrease in carotenoid (vibrational bands around 1,523 and 1,541 cm^−1^) was consistently seen in the symptomatic compared to the asymptomatic tissue. Using Raman spectroscopy, Rys et al. (2014) reported an increase in carotenoid bands during Obuda pepper virus infection at 48 h post-infiltration (hpi); however, the measurement by traditional spectrophotometric methods did not show significant changes in total carotenoid content. This discrepancy was attributed to a qualitative rather than quantitative change in total carotenoids or to the ability of Raman spectroscopy to detect different sets of carotenoids in comparison to the spectrophotometric method.

We have previously used Raman spectroscopy to identify Arabidopsis plants suffering from the early stages of nitrogen deficiency (Huang et al., 2020). In rice, a hand-held Raman spectrometer showed an accurate presymptomatic diagnosis of N, P, and K deficiencies, as well as a medium and high salinity stresses, with a hydroponic condition (Sanchez et al., 2020a). We seek to extend this noninvasive method for the diagnosis of plants that exhibit early host responses to pathogen infection.

Plants have evolved a multilayer defense system to combat pathogens (Li and Staiger, 2018). The first layer of the plant immune system involves pathogen perception by the recognition of conserved pathogen-associated molecular patterns (PAMPs) by pattern recognition receptors (PRRs) present on the plant cell surface. These plasma membrane-localized PRRs are mainly found as receptor kinases or receptor-like proteins and, upon binding of the PRRs, initiate signaling of an innate immune pathway referred to as PAMP-triggered immunity (PTI) (Ausubel, 2005; Abdul Malik et al., 2020). PAMP-triggered immunity (PTI) causes rapid plant defense responses, including calcium ion flux, reactive oxygen species (ROS) burst, the activation of the MAPK cascades, the expression of defense-related genes, and callose deposition (Jones and Dangl, 2006; Luna et al., 2011; Bigeard et al., 2015). Two of the most studied PRRs are flagellin-sensing 2 (FLS2), which senses a conserved 22-amino acid sequence of flagellin (flg22), and elongation factor Tu (EF-Tu) receptor (EFR), which recognizes a conserved 18-amino acid peptide (elf18) of the EF-Tu (Zipfel et al., 2004, 2006; Chinchilla et al., 2006). The signaling mechanisms of these two PRRs have been well studied, and the key components have been identified (Sun et al., 2013; Yu et al., 2017; Peng et al., 2018; Saijo et al., 2018; Wang et al., 2018).

To see if we can employ Raman spectroscopy to detect early changes in cellular metabolites following pathogen–plant interaction, we used the elicitors flg22 and elf18 to trigger PTI in Arabidopsis plants. Raman spectra were taken in the leaf region proximal to the mock- or elicitor-infiltrated site, and the difference Raman spectrum revealed several peaks along with carotenoids that showed significant changes at 24 hpi. To quantify the early PTI response using Raman spectroscopy, an Elicitor Response Index (ERI) was developed whereby a higher ERI value indicates a more significant elicitor-induced immune response. Investigations of several PTI-related mutants produced the expected results, substantiating the reliability and reproducibility of this method. This approach was further validated by using pathogen studies in Arabidopsis and the vegetable crop, Choy Sum (*Brassica rapa v.parachinenesis*). We suggest that the noninvasive optical method described in this study can be used to identify the early infection of Arabidopsis and field crops.

## Materials and Methods

### Plant Growth and Elicitor/Pathogen Infiltration

*Arabidopsis thaliana* (Col-0) wild type (WT) and mutants including *fls2* (salk_062054), *efr-2* (salk_068675), *bak1* (salk_116202), *bik1* (salk_005291), *rbohd* (salk_083046), *pub12/13* (Zhou et al., 2018), and *pub25/26* (Wang et al., 2018) were used. Ten-day-old seedlings grown on soil were transferred to a growth room at 70% humidity and 21°C with a 10-h-light/14-h-dark photoperiod (short-day condition). Light intensity was 100 μmol/m^2^s^−1^. Five- to six-week-old plants with about 17–18 leaves were used. Flg22 contains the 22-amino acid sequence QRLSTGSRINSAKDDAAGLQIA corresponding to a conserved motif of the amino terminus of flagellin from *Pseudomonas* species. Elf18 contains the sequence AcSKEKFERTKPHVNVGTIG corresponding to the conserved N-terminal 18-amino acid peptide of the bacterial protein EF-Tu. These peptides (Axil Scientific Pte Ltd., Singapore Science Park II, Singapore) were dissolved in sterile H_2_O to a concentration of 5 mM, and aliquots of 100 μM stock solution were stored at −20°C. 3-hydroxydecanoic acid (Sigma, St. Louis, MO, USA, H3648) (Kutschera et al., 2019) was dissolved in ethanol to a concentration of 5 mM, and Chitin, GlcNacβ1-4[GlcNacβ1-4GlcNAc]5GlcNAc (ELICITYL, Crolles, France, GLU437) was dissolved in water to a concentration of 5 mM. Aliquots of 100 μM stock solutions were stored at −20°C.

An overnight culture of *Pseudomonas syringae pv. Tomato* DC3000 (*Pst* DC3000) (Rufián et al., 2019) was suspended in 10 mM MgCl_2_ to approximately 5 × 10^5^ cfu/ml (OD_600_ = 0.001). About 50 μl of either the bacterial suspension or 10 mM MgCl_2_ (mock) was infiltrated on the abaxial side of Arabidopsis leaf by a needleless syringe. Commercial Choy Sum (*B. rapa v. parachinensis*) seeds were grown in growth chambers at 25°C under a 16-h-ligh/8-h-dark photoperiod with 75% humidity. Two-week-old Choy Sum plants were inoculated with the *Xanthomonas campestris py. campestris (Xcc)* isolate (ATCC33913). The *Xcc* strain was grown in yeast extract, glucose, and calcium carbonate (YGC) medium at 28°C. Harvested bacterial cells were resuspended in YGC medium to 1 × 10^8^ cfu/ml. Using a needleless syringe, the abaxial side of the fourth leaf of each plant was inoculated with 200 μl of *Xcc* suspension on both sides of the central leaf vein near the leaf margins. The leaves of separate plants inoculated with 200 μl of sterile media served as the mock control.

### Custom-Built Raman Spectroscopy System and Spectral Data Analysis

The Raman spectroscopy system with an 830 nm excitation wavelength was previously described (Huang et al., 2020). The Raman shift axis was calibrated using a polystyrene sample (Creely et al., 2005), and the Raman spectra were recorded in the range of 400–1,700 cm^−1^. The 830 nm excitation wavelength has the advantage of deeper penetration depth and generating a comparatively lower fluorescence background signal from the leaf tissue, resulting in spectra with a higher signal-to-noise ratio (SNR). The preprocessing of the Raman spectra consisted of cosmic ray removal and smoothing with the Savitzky–Golay filter function (MATLAB, Inc., Natick, MA, USA) with the fifth-order polynomial and frame length of 11. The Raman spectra presented in this study were obtained after removing any residual fluorescence background using a polynomial subtraction method with nonnegative constraints (Lieber and Mahadevan-Jansen, 2003).

**Figure 1:**
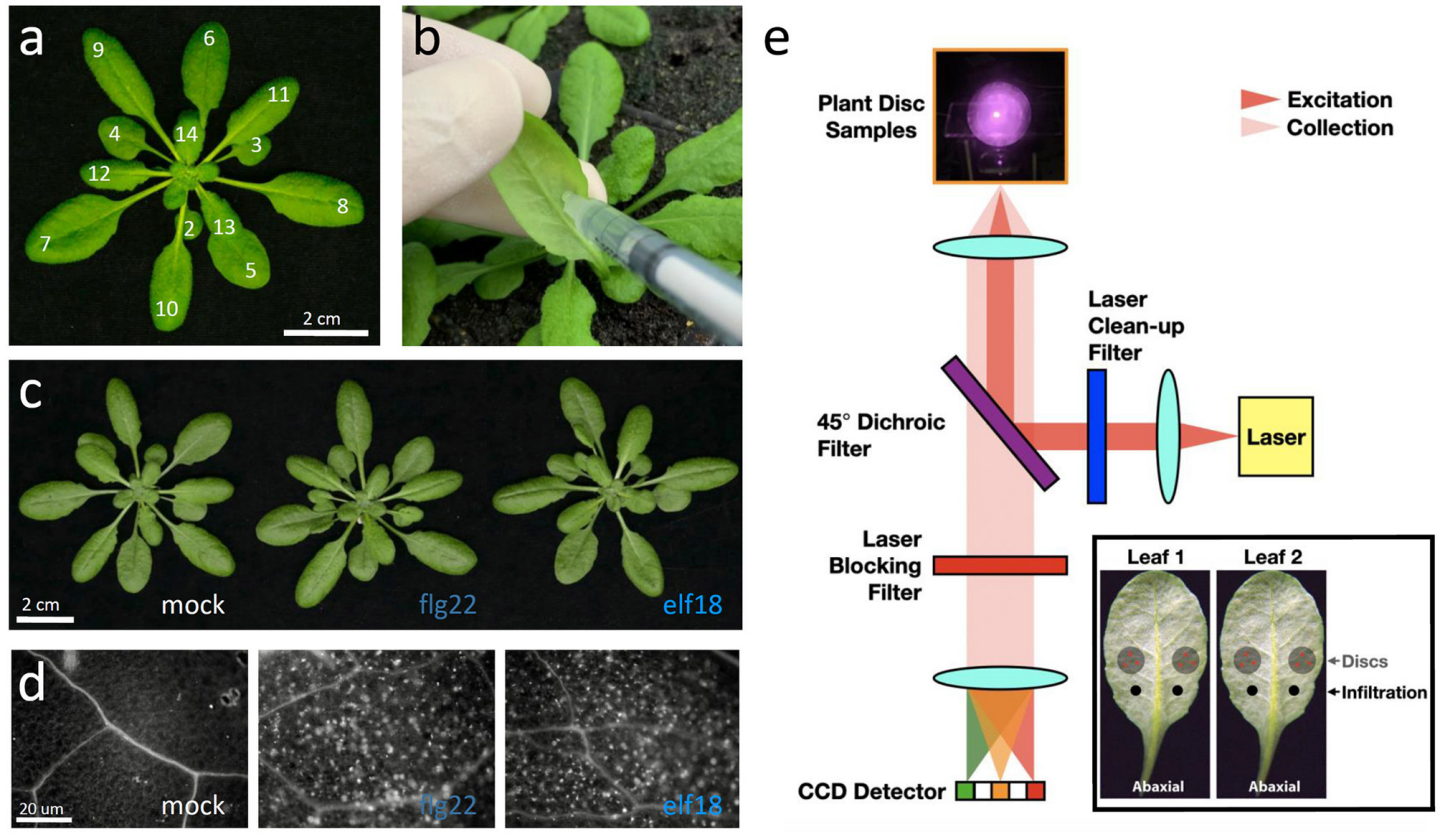
Experimental procedures for the Raman spectroscopic analysis of elicitor-treated plants. **(a)** A 5-week-old *Arabidopsis thaliana* plant with leaves numbered according to the developmental stage. Leaf 1 is the first true leaf that started to emerge after the cotyledons. Scale bar = 2 cm. **(b)** The abaxial (bottom) side of the leaf to be syringe-infiltrated. Leaf was inoculated by infiltration with a blunt syringe. **(c)** Images of plants 24 h post-infiltration (hpi). Scale bar = 2 cm. **(d)** Images of callose deposits in aniline-blue-stained leaves that were previously infiltrated with mock (H_2_O), 1 μM flg22 or elf18 for 24 h. Scale bar = 20 μm. **(e)** Schematic of a custom-built NIR Raman spectroscopy used to detect secondary metabolites in plants. An insert shows the infiltrated sites and the leaf discs taken from the proximal region (PR) of the sites for analysis.

**Figure 2:**
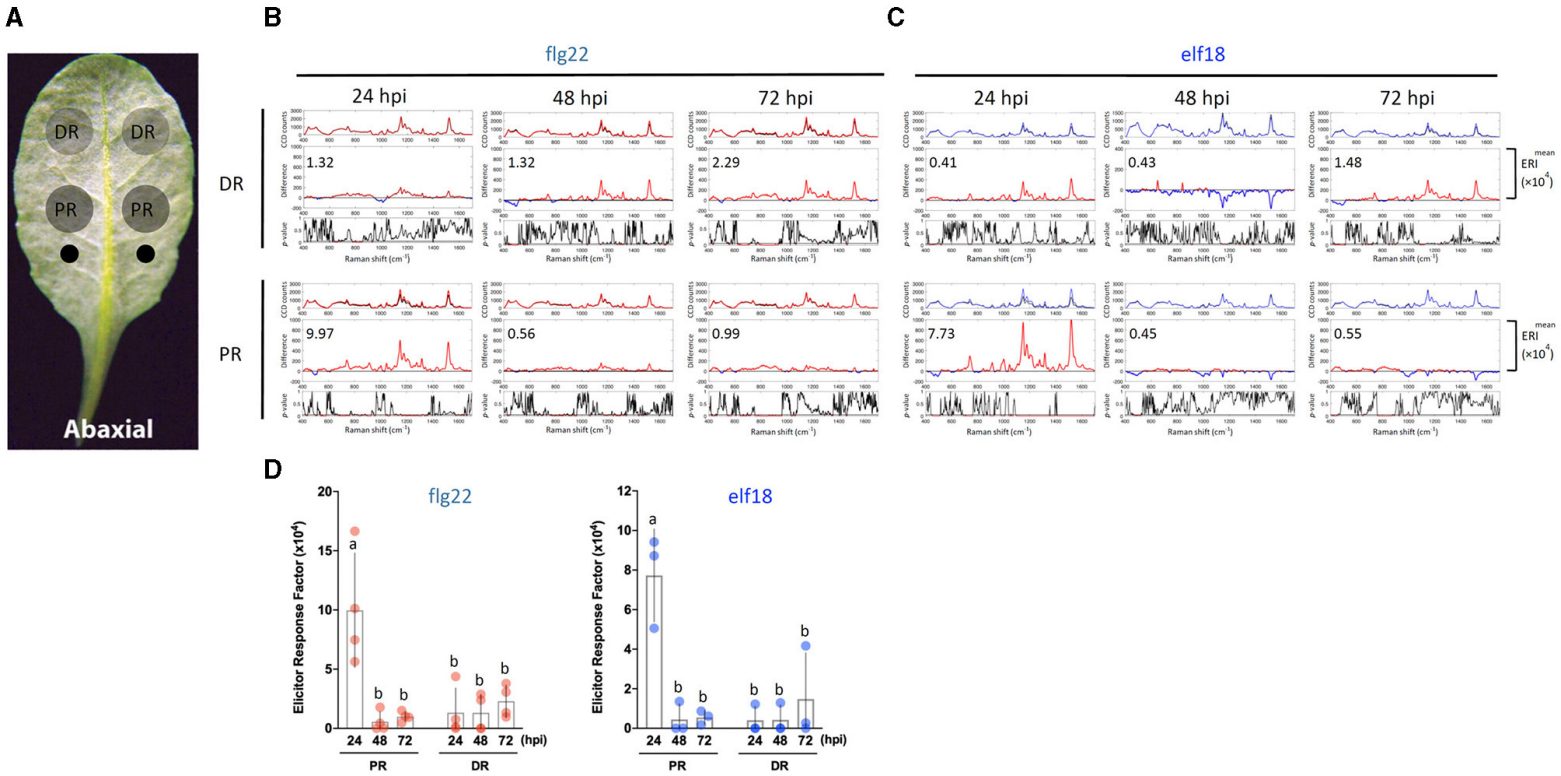
Effects of sample location and elicitor treatment time on changes in Raman spectra. **(A)** The abaxial (bottom) side of a leaf is to be infiltrated with an elicitor. Elicitor (1 μM) was infiltrated with a needleless syringe at two locations (indicated as two small circles) nearest to the petiole. Leaf samples were taken from the PR and DR at 24, 48, and 72 hpi and analyzed by Raman spectroscopy. **(B,C)** A group of three panels was shown for either the DR or PR sample. Upper panel: mean spectra of control (black) and elicitor-treated samples (red, flg22 and blue, elf18). Middle panel: Difference spectrum between the mean spectrum of the elicitor-treated sample vs. that of the mock-treated sample. Red indicates that the difference is a positive value, whereas blue indicates a negative value. The number inside the panel indicates the average Elicitor Response Index (ERI, ×10^4^) value obtained from four **(B)** or three **(C)** independent experiments. Lower panel: *t*-test was used to evaluate statistically significant differences between elicitor-infiltrated and H_2_O-infiltrated (mock) samples and the results were expressed as *p*-values. Red indicates *p* < 0.05. **(D)** Changes in ERI after the time of infiltration. Left, flg22 (red spots), *n* = 4 independent biological samples; Right, elf18 (blue spots), *n* = 3 independent biological samples. PR, proximal region; DR, distal region. The results represent the average values of three to four independent biological experiments with SD. Bars with different letters above are significantly different according to Fisher's least significant difference test (*p* < 0.05).

For Arabidopsis, two plants from each independent biological sample were investigated (Huang et al., 2020). The seventh or eighth leaf of 5–6-week-old plants was used for infiltrations (Figure 1). At the designated time after infiltrations, leaf discs (6 mm diameter) were excised with a puncher from the regions proximal to the infiltrated sites (Figure 2A). Two leaf discs were obtained from each elicitor-treated leaf, one from the left and the other from the right side of the central leaf vein. A total of four leaf discs were analyzed for each genotype per experiment. The leaf discs were placed with a drop of water between a fused silica slide of thickness 0.5 mm and a fused silica coverslip, which was 100 μm thick. Three locations were measured in each leaf disc, with five Raman spectra acquired per location and an exposure time of 10 s per spectrum. Thus, an average Raman spectrum of an independent biological sample was obtained from the mean of a total of 60 Raman spectra. The experiments were repeated with three to four independent biological samples.

For Raman spectral acquisitions in Choy Sum, the two leaf discs from a single leaf were excised from the proximal regions (PRs) of the infiltrated site at 24 and 48 h after infiltration. Three locations were measured in each leaf disc, with five Raman spectra acquired per location and an exposure time of 10 s per spectrum. Thus, an average Raman spectrum of one independent biological sample for each time point was obtained from the mean of a total of 30 Raman spectra. Three biological replicates for each time point were performed for both *Xcc* and mock infiltration.

### RNA Extraction and Gene Expression Analysis by Reverse Transcription-Quantitative PCR

After mock or elicitor treatments, the leaf samples were collected at time periods indicated in each experiment (0.5, 1, 3, 6, and 24 h) and immediately frozen in liquid nitrogen. Total RNA was extracted from the entire infiltrated leaf using the RNeasy plant mini kit (74104; Qiagen, Hilden, Germany) with DNase I (79254; Qiagen, Hilden, Germany) treatment. About 1 μg of RNA was used for complementary DNA (cDNA) synthesis, and reverse transcription-quantitative PCRs (RT-qPCRs) were performed as described (Park et al., 2018) on the following genes. Immune response genes—*WRKY53* (At4g23810), early senescence WRKY networking regulator; *WRKY29* (At4g23550); *WRKY30* (At5g24110); *ZAT12* (At5g59820), ZINC FINGER OF ARABIDOPSIS THALIANA12; *PR1* (At2g14610), PATHOGENESIS-RELATED GENE1; *PDF1.2* (At5g44420), PLANT DEFENSIN; *MPK3* (At3g45640), Mitogen-activated Protein Kinase3; *SAND* (At2g28390), MONENSIN SENSITIVITY1 (MON1) a zinc finger protein, *PMR4* (At4g03550), *PAD3* (At3g26830), *MPK3* (At3g45640), and *MYB51* (At1g18570). Tryptophan-metabolism related genes. *MYB12*2 (At1g74080), *MYB51* (At1g18570), *MYB34* (At5g60890), *CYP79B2* (At4g39950), *CYP79B3* (At2g22330), *CYP83B1* (At4g31500), *CYP71A13* (At2g30770), *CYP71B15*/*pad3* (At3g26830) Carotenoid biosynthetic genes-*PSY*, phytoene synthase (At5g17230); *PDS*, phytoene desaturase (At4g14210); *Z-ISO*, z-carotene isomerase (At1g10830); *ZDS*, z-carotene desaturase (At3g04870); *CRTISO*, carotenoid isomerase (At1g06820); ε*LCY*, Lycopene ε-cyclase (LUT2, At5g57030); β*LCY*, Lycopene β-cyclase (At3g10230); *CYP97A3*, (LUT5, At1g31800); *CYP97C1*, (LUT1, At3g53130); *CYP97B3*, (At4g15110); *BCH1*, β-carotene hydroxylase 1 (At4g25700); *BCH2*, β-carotene hydroxylase 2 (At5g52570); *CCS*, COPPER CHAPERONE FOR SOD1(At1g12520); *ZEP*, Zeaxanthin epoxidase (ABA1, At5g67030); *NPQ*, NON-PHOTOCHEMICAL QUENCHING1 (At1g08550), *NXS*, neoxanthin synthase (ABA4, At1g67080); *NCED3*, 9-cis-epoxycarotenoid dioxygenase (At3g14440); *ABA2*, ABA DEFICIENT 2 (At1g52340); *AAO3*, ABSCISIC ALDEHYDE OXIDASE3 (At2g27150); *CCD1*, carotenoid cleavage dioxygenase1 (At3g63520); *CCD4*, At4g19170; *CCD7*, At2g44990; *CCD8*, At4g32810; *D27*, β-carotene isomerase (At1g64680); *CYP711A*, (At2g26170). The expression of the Arabidopsis *Act2* (At3g18780) was used as an internal control. Primer pairs for RT-qPCR were designed using Primer-BLAST (https://www.ncbi.nlm.nih.gov/tools/primer-blast/index.cgi) or manually by eye. All RT-qPCR primers are listed in Supplementary Table 1.

### Elicitor-Induced Callose Deposition

The callose assay was performed as described by Ton et al. (2005). Callose-mediated fluorescence was visualized using a DAPI filter set (an excitation filter 365 nm; a dichroic mirror 395 nm; and an emission filter 397 nm) of an Axio Imager M2 microscope (Zeiss, Oberkochen, Germany). Images collected with a photometric CoolSNAP HQ2 camera system (Roper Scientific Germany, Planegg, Germany) were acquired by an x100 lens using the MetaMorph software (Version 7.7.0.0; Molecular Devices, Sunnyvale, CA, USA). Callose foci within the frame of a single image (magnification ×100; 5,655 μm^2^ per region) were quantified using the ImageJ software (http://rsb.info.nih.gov/ij/, Schneider et al., 2012). For each experiment, the number of callose deposits was measured in two plants with about four to five leaf regions surveyed per plant. This provided about 8–10 data points per experiment. The experiment was repeated with three independent biological samples. For Choy Sum, callose deposition was visualized in three independent biological samples at 24 and 48 hpi.

### Total Carotenoid Extraction for Spectrophotometric Analysis

Total carotenoids were extracted and analyzed as described with minor modifications (Sumanta et al., 2014). Briefly, 30 mg of the fresh leaf sample was homogenized in a tissue homogenizer with 600 μl of methanol solvent. After centrifugation at 10,000 × g for 15 min at 4°C, 0.1 ml of the supernatant was mixed with 0.9 ml of methanol. The mixture was analyzed for photosynthetic pigment content by a spectrophotometer (TECAN, Männedorf, Switzerland, The spark multimode microplate reader) according to Sumanta et al. (2014). For each experiment, the quantification of total carotenoids was measured in three to five infiltrated plants per experiment. The quantification of carotenoids was expressed as the mean value of 15–20 different leaf extracts in four replicated experiments.

## Results

### Flg22 and elf18 Elicit Robust PTI Response

We used flg22 and elf18 to induce plant innate immunity responses without related pathogenic symptoms (Bektas and Eulgem, 2015). To reduce variability between biological samples, Arabidopsis WT (Col-0) and mutant plants were grown under short-day conditions for about 5–6 weeks with about 17–18 leaves (Farmer et al., 2013; Kurenda et al., 2019) (Figure 1a). The abaxial side of leaf seventh or eighth was infiltrated with a needleless syringe containing (50 μl) water, 1.0 μM flg22, or 1.0 μM elf18 (Figure 1b). At 24 hours post-infiltration (hpi), no pathological symptoms were seen at the infiltration sites in response to the PAMP nor the slight wounding caused by the syringe infiltration (Figure 1c). However, compared to mock infiltration, the infiltration of either induced the expression of PTI-specific marker genes but with different kinetics (Supplementary Figure 1). Some early responsive genes (*WRKY30, MPK3, MYB51, CYP79B2*, etc.) showed increased expression as early as 3 hpi, whereas the expression of other genes (*PR1, PDF1.2, ZAT12*, and *MYB122*) increased only at 6 hpi. The expression of most activated genes returned to basal levels by 24 h (Supplementary Figure 1) although several genes (*PR1, WRYK29*, and *WRKY53)* still maintained high expression levels at this time point. One day after infiltration, callose deposition in the elicitor-infiltrated leaf was more significantly induced than that in the mock-infiltrated control leaf (Figure 1d), which was consistent with the increased expression of genes related to callose synthesis (Supplementary Figure 1). The gene expression results and the callose deposition data together confirmed that, under our experimental conditions, both flg22 and elf18 induced a rapid and robust innate immunity response in the infiltrated leaves.

### Analysis of the PTI Response by Raman Spectroscopy: Development of ERI

We used a custom-built near IR excitation Raman instrument (Figure 1e) to measure the Raman spectra from mock- or elicitor-infiltrated leaf samples (Figure 2). To quantify the PTI response using Raman spectroscopy, we developed an ERI as described as follows (Supplementary Figure 2):

**Step 1**: After preprocessing, as described in the section “Materials and methods,” the mean of 60 Raman spectra from each individual biological sample was obtained in the Raman shift spectral range of 400–1,700 cm^−1^.
**Step 2**: The difference of the mean spectra, as obtained in Step 1, between elicitor-treated and mock control-treated samples were subsequently derived to highlight the different Raman spectral regions with positive values.
**Step 3**: A *p*-value plot was obtained using a *t*-test to evaluate the statistical significance of the difference Raman spectra obtained in Step 2. The *p*-value of < 0.05 was noted in the difference Raman spectral regions. The *p*-value plot was corrected by including an estimation of the positive false discovery rate (pFDR) and applying the multiple-hypothesis testing principle.
**Step 4**: The area under the curve of the difference Raman spectral region, which has a positive value and also represents the corrected *p*-value of < 0.05 of Step 3, is defined as the ERI, which we used to measure the level of PTI response. A higher ERI value indicated a higher level of the elicitor-induced immune response.
**Step 5:** All the positive spectral regions contributing toward ERI were tabulated.

### Effects of Elicitor Concentration on PTI Response Measured by Raman Spectroscopy

Because a robust induction of PTI marker genes was seen with 1 μM flg22 or elf18 (Seo et al., 2019), we use this elicitor concentration to perform a time-course experiment on leaf regions PR or distal (DR) from the infiltrated sites (Figures 2A–C). For flg22, the highest Raman shift spectrum and a high ERI value were obtained at PR 24 hpi. The ERI value decreased to almost zero at 48 and 72 hpi (Figures 2B,D). Low ERI values were obtained in DR, which gradually increased with time at 48–72 hpi indicating signal transmission from PR to DR. For elf18, Raman shift signals were also detected at 24 hpi in PR with high ERI values, which decreased to almost zero at 48–72 hpi. A lower ERI value was seen in DR (Figures 2C,D). Based on these results, for subsequent experiments, we chose to analyze the Raman spectra at PR 24 hpi with an elicitor concentration of 1.0 μM.

### Raman Spectral Analysis of PAMP-Signaling Mutants Defective in Positive or Negative Regulatory Function

**Figure 3:**
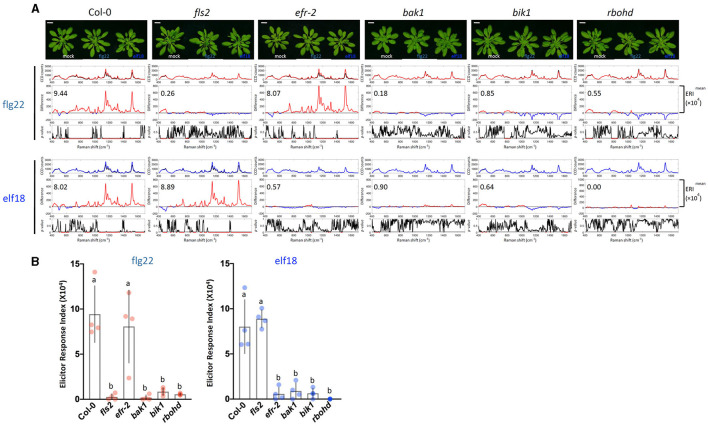
Raman spectroscopic analysis of wild type (WT) and mutant plants. **(A)** Raman spectra of WT Col-0 and mutant plants (*fls2, efr-2, bak1, bik1*, and *rbohd*) at 24 h after flg22 and elf18 treatment and mock control. Top panel: Images of plants 24 hpi. For each elicitor experiment (flg22, red and elf18, blue), a group of three panels was shown. Scale bar = 2 cm. Upper panel: a mean plot of all 60 spectra, taken from control (black) or elicitor-treated samples (red); Middle panel: the difference spectrum between the mean spectrum of the elicitor-infiltrated sample and that of the H_2_O-infiltrated (mock) sample. Red indicates that the difference is a positive value, whereas blue indicates a negative value. The number inside the panel indicates the average ERI (×10^4^) value obtained from four independent experiments. Lower panel: *t*-test was used to evaluate statistically significant differences between elicitor-infiltrated and H_2_O-infiltrated (mock) samples and the results were expressed as *p*-values. Red indicates *p* < 0.05. Plants: Col-0, WT *A. thaliana*; *fls2, FLAGELLIN-SENSING2* (At5g46330); *efr-2, EF-Tu receptor* (At5g20480); *bak1, BRASSINOSTEROID INSENSITIVE-ASSOCIATED KINASE1* (At4g33430); *bik1, BOTRYTIS-INDUCED KINASE1* (At2g39660); and *rbohd, NADPH/respiratory burst oxidase protein D* (At5g47910). **(B)** ERI of WT and mutants treated with either flg22 or elf18. The results show average ERI values along with SD. Bars with different letters above are significantly different according to Fisher's least significant difference test (*p* < 0.05). Left: flg22 (red spots); Right, elf18 (blue spots). *n* = 4 independent replicates.

To confirm that the Raman spectra changes obtained by the elicitors were indeed caused by PTI signaling, we first analyzed the Raman spectra of a well-characterized mutant of the PTI pathway. Arabidopsis plants possess two PAMP receptors. The FLS2 receptor specifically recognizes flg22, whereas the EFR receptor is specific for elf18 (Chinchilla et al., 2006; Zipfel et al., 2006). Figure 3 shows the response of WT plants to both flg22 and elf18 with similar ERI values. By contrast, the *fls2* mutant was refractory to flg22 but remained responsive to elf18, and similarly, the *efr-2* mutant responded to flg22 but not to elf18 (Figure 3). These results confirmed that the immune response is specific to the elicitor used. We also measured the Raman spectra of mutants, including *bak1, bik1*, and *rbohd*, that are blocked in the PAMP signal transduction downstream of receptor activation (Figure 3). Previous publications have reported that the following mutants of the PAMP-signaling pathway, *fls2, efr-2, bak1, bik1*, and *rbohd*, have significantly reduced callose deposition after elicitor treatment (Lu et al., 2009; Zhang et al., 2010; Jaillais et al., 2011; Belkhadir et al., 2012; Daudi et al., 2012; Chaudhary et al., 2014; Smith et al., 2014). Callose deposition patterns and expression changes of marker genes of these mutants confirmed these previous results and verified their positive regulatory role in PTI response (Supplementary Figures 3, 4A). All these mutants did not show any significant changes in Raman spectra after elicitor treatment and hence had low ERI values (< 1 × 10^4^) (Figure 3).

**Figure 4:**
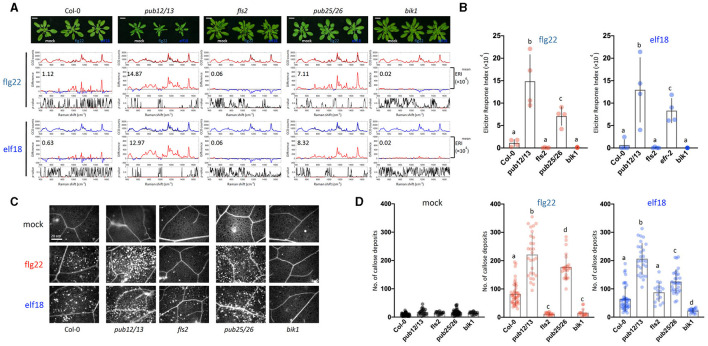
Raman spectroscopic analysis of WT and *pub12pub13* and *pub25pub26* double mutants. **(A)** Top panel: images of plants 10 hpi. For each elicitor experiment (flg22, red and elf18, blue), a group of three panels was shown. Raman spectra were acquired at 10 hpi with 1.0 μM flg22 (red peak) and elf18 (blue peak) treatment and mock control (black peak). Upper panel: a mean plot of all 60 spectra, taken from control (black) or the elicitor-treated samples (red, flg22 and blue, elf18); Middle panel: the difference spectrum between the mean spectrum of the elicitor-infiltrated sample and that of the H_2_O-infiltrated (mock) sample. Red indicates that the difference is a positive value, whereas blue indicates a negative value. The numbers inside the panel indicate average ERI values. Lower panel: a *t*-test was used to evaluate statistically significant differences between elicitor-infiltrated and H_2_O-infiltrated (mock) samples and the results were expressed as *p*-value. Red indicates *p* < 0.05. Plants: Col-0, WT *A. thaliana*; *pub12pub13*, U-box E3 ubiquitin ligase PUB12/13 (At2g28830 and At3g46510, respectively); *fls2*, FLAGELLIN-SENSING2 (At5g46330); *pub25pub26*, U-box E3 ubiquitin ligase PUB25/26 (At3g19380 and At1g49780, respectively); *bik1*, BOTRYTIS-INDUCED KINASE1 (At2g39660). Scale bars = 2 cm. **(B)** ERI of WT and mutants treated with either flg22 or elf18. The results show mean ERI values along with SD. Bars with different letters above are significantly different according to Fisher's least significant difference test (*p* < 0.05). Left: flg22 (red spots); Right, elf18 (blue spots). *n* = 4 independent biological replicates. **(C)** Representative images of callose deposits (bright spots) in the treated samples; Scale bars = 20 μm. **(D)** Quantification of callose deposits in WT (Col-0), *pub12/13, fls2, pub25/26*, and *bik1* mutant leaves. The number of callose fluorescence spots was used to determine the number of callose deposits. Left: mock (black spots); Middle: flg22 (red spots); Right, elf18 (blue spots). The values shown are mean ± SD of 25–30 surveyed leaf regions. For each experiment, the number of callose deposits was measured in two infiltrated plants with about four to five leaf regions surveyed per plant. The value for each leaf region gives the number of callose deposits per 5,996 μm^2^. This gave a total of about 8–10 data points per experiment. The experiment was repeated with three independent biological samples. The graph shows an average value with *n* = 25–30. Bars with different letters above are significantly different according to Fisher's least significant difference test (*p* < 0.05).

Following the activation of the PAMP receptor, the PTI-signaling pathway is fine-tuned by downregulation to prevent excessive or prolonged activations that would be deleterious to the host plants. One common mechanism of signaling desensitization is by the polyubiquitin-mediated proteolysis of signaling components, and several such components have been identified. The FLS2 receptor itself is known to be poly-ubiquitinated by PUB12/13 (Lu et al., 2011; Zhou et al., 2015), and the coactivator BIK1 is similarly modified by PUB25/26 (Wang et al., 2018). Therefore, both PUB12/13 and PUB25/26 are the negative regulators of the PTI response, and the mutants that are deficient in these E3 ligases should display hyperresponsiveness to elicitors. In a time-course experiment, we found that the *pub* mutants (*pub12/13* and *pub25/26*) indeed showed increases in gene expression levels as early as 0.5 or 6 h after 1 μM elicitor treatment when little response was detected with WT (Supplementary Figure 4B). Based on the earlier gene expression kinetics, we decided to measure changes in Raman spectra at 10 hpi. Figure 4 shows that at 10 h after flg22 treatment, the WT ERI value was only 1.12 × 10^4^ as compared to an ERI value of 9.44 × 10^4^ at 24 hpi (Figure 3A). Similarly, in the case of elf18, the ERI value at 10 hpi was 0.63 × 10^4^ compared to 8.02 × 10^4^ at 24 hpi (Figures 4A,B for 10 h and Figures 3A,B for 24 h). These results suggest that in WT plants the PAMP signaling pathway was only slightly activated at 10 h, and full activation would require 24 hpi. By contrast, at 10 hpi of either elicitor *pub12/13* displayed an ERI value about 13–21 times higher than WT values, and for *pub25/26*, about 6.4–13.3 times higher (Figure 4B). These results showed that the hyper-responsive nature of the *pub* mutants to elicitors can be confirmed by Raman spectroscopy. Interestingly, higher ERI values were found in *pub12/13* targeting an FLS2 receptor compared to those of *pub25/26* targeting a downstream regulator, BIK1, in the flg22/elf18 signaling pathway (Figure 4B).

The ERI values were evaluated based on the Raman spectrum analysis at 24 spots in 4 individual plants (2 leaf discs per plant) with 4 independent experiments giving a range of numbers between the high (>5 × 10^4^) and low (0.0–1.0 × 10^4^) level according to the PTI response (Figure 4B). These results confirmed the accuracy of the ERI indexing based on Raman spectral assay for determining PTI responses. Consistent with these analyses, the *pub12/13* and *pub25/26* mutant plants showed more callose deposits than WT and the two control mutants, *fls2* and *bik1* (Figures 4C,D).

### Elucidation of Metabolic Changes Contributing Toward a High ERI Value at 24 hpi

**Figure 5:**
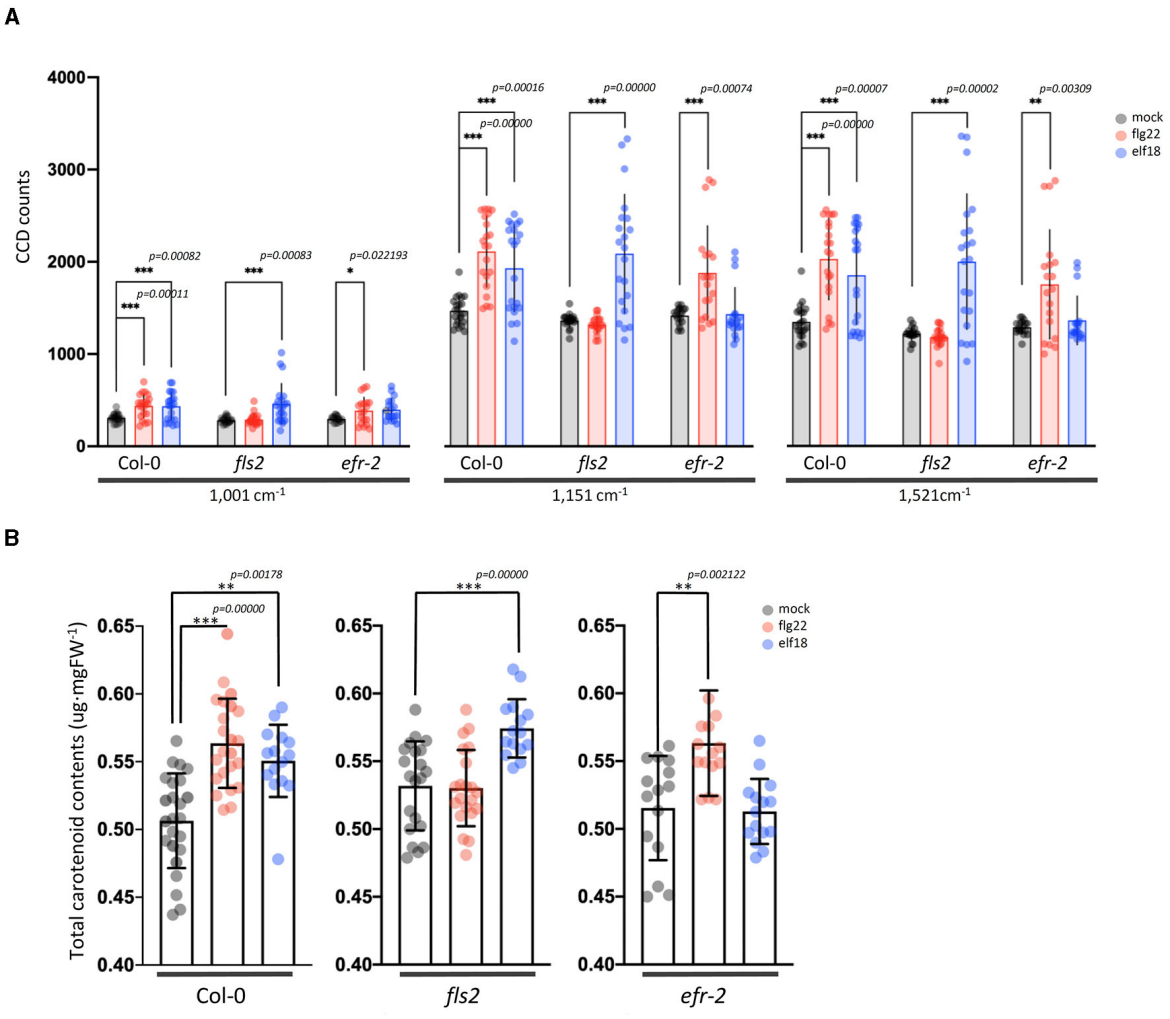
Changes in carotenoid levels in Arabidopsis leaf samples treated with an elicitor for 24 h. **(A)** Raman spectra of carotenoids from the leaves with mock and elicitor treatment for 24 h. Raman shifts at 1,001, 1,151, and 1,521 cm^−1^ are attributed to carotenoids. *n* = 3 independent biological samples. **(B)** Changes of total carotenoids levels in Arabidopsis leaves after treatment with mock control, flg22, and elf18 for 24 h (24 hpi). Carotenoid levels were determined as described in the “Materials and methods” section. The values shown are mean ± SD of the extracted total carotenoids. For each experiment, the quantitation of total carotenoids was measured in three to five infiltrated plants per experiment. The experiment was repeated with four independent biological samples, and this gave a total of about 15–20 data points. ** and *** significantly different from the mock control at *p* < 0.001 and *p* < 0.0001 (*t*-test), respectively.

Elicitor Response Index presents a quantitative and holistic measure of changes in the Raman spectra in elicitor-treated samples relative to mock control samples. In this case, we consider changes in the peak intensities for individual vibrational bands and consider the metabolites that give rise to these changes. For WT plants at 24 hpi, the majority of the Raman peaks that exhibit changes are common to both elicitor responses, flg22 and elf18. The difference spectra with annotated peaks are shown in Supplementary Figure 5. Table 1 summarizes the relevant vibrational bands (*p* < 0.05) for various elicitors, and the peak assignments are based on previously published reports. An increase in peak intensity at vibrational bands originating from carotenoids, pectin, phenylpropanoids, cellulose, lignin, and proteins is observed, which contributes toward a high ERI value seen at 24 hpi. Among these, the most prominent increase in peak intensity was associated with carotenoids. WT plants showed a significant increase in peak intensities at around 1,001, 1,151, and 1,521 cm^−1^ at 24 hpi for both the elicitors (Figure 5A). Peak intensities at 1,151 and 1,521 cm^−1^ were also increased in the case of receptor mutants treated with a heterologous elicitor (Figure 5A). These strong Raman peaks are attributed to carotenoids, and it is these molecules that exhibit the largest change in concentration. Previous reports have attributed peaks in the 1,100–1,200 and 1,400–1,600 cm^−1^ Raman shift regions to carotenoids (Payne and Kurouski, 2021). These peaks are due to in-phase carbon-carbon stretching vibrations of the main polyene chain with the exact Raman shift locations of these peaks being dependent on the polyene chain length. These results suggest that an early event in plant PTI response is likely an increase in carotenoid concentration, which contributes significantly toward a high ERI value. To confirm the Raman spectral results with regard to an increase in carotenoid concentration, we extracted the photosynthetic pigments (chlorophyll a, b, and carotenoids) from the treated leaves and measured changes in their content directly. The treatment of WT with elicitors produced a quantitative increase of more than 5% of total carotenoids. In the case of *fls2* and *efr-2* mutants, the carotenoid content was increased by 10% when challenged with heterologous elicitors (Figure 5B). The results are supported by increased expression of genes related to carotenoid biosynthesis at early time points of elicitor treatment (Supplementary Figure 6).

**Table 1:** Significant vibrational bands and their assignments that contributes to the high ERI value at 24 hrs following flg22 and elf18 treatment respectively.

**Raman peak (cm^**−1**^)**	**Assignment**	**Molecular bond**
**flg22**		
742.3	Pectin	γ(C–O–H) of COOH
		ring br. mode (aromatics) [##]
789.7*	Chlorophyll a, phosphodiester	(OPO) sym [#]
914	Leucine, cellulose, lignin	(C–C), (C–N) stretching [$]
1001	Phenylalanine, carotenoids	ν3 (C–CH_3_ stretching), phenylalanine ring stretching mode [##]
1107	Leucine, carbohydrates	(C–C), (C–N) stretching
		(C–OH), (C–O–H) stretching [$]
1151	Carotenoid	C–C stretching; v(C–O–C) [##]
1180	Carotenoid	dCH, u (pyr half-ring)_as_ [$$]
1211	Phenylalanine, tryptophan	u (C–C_6_H_5_), u_18_ (dC_m_H) [$$]
1276	Carotenoid	Amide III [#]
1318	Cellulose/lignin/protein	dCH_2_ bending [##]
1381	Proteins, nucleic acids	dCH_3_ symmetric [#]
1433	Proteins, lipids	CH_2_ scissoring [#]
1521	Carotenoid	–C=C– (in-plane) [#]
1552	Amide II, proteins: tyrosine, tryptophan, chlorophyll a	Tryptophan, u (C=C) [#]
1608	Lignin	u (C–C) aromatic ring + s(CH) [##]
**elf18**		
742.3	Pectin	γ(C–O–H) of COOH
		ring br. mode (aromatics) [##]
914	Leucine, cellulose, lignin	(C–C), (C–N) stretching [$]
1001	Phenylalanine, carotenoids	ν3 (C–CH_3_ stretching), phenylalanine ring stretching mode [##]
1065*	Lipids	Skeletal C–C stretch of lipids [#]
1107	Leucine, carbohydrates	(C–C), (C–N) stretching
		(C–OH), (C–O–H) stretching [$]
1151	Carotenoid	C–C stretching; *v*(C–O–C) [##]
1180	Carotenoid	dCH, u (pyr half-ring)_as_ [$$]
1211	Phenylalanine, tryptophan	u (C–C_6_H_5_), u_18_ (dC_m_H) [$$]
1276	Carotenoid	Amide III [#]
1318	Cellulose/Lignin/Protein	dCH_2_ bending [##]
1381	Proteins, nucleic acids	dCH_3_ symmetric [#]
1433	Proteins, lipids	CH_2_ scissoring [#]
1521	Carotenoid	–C=C– (in-plane) [#]
1549	Amide II, proteins: tyrosine, tryptophan, chlorophyll a	Tryptophan, u (C=C) [#]
1608	Lignin	u (C–C) aromatic ring + s(CH) [##]

“*” *denotes vibrational bands, which were specific to each elicitor*.

“#” *denotes reference Movasaghi et al. (2007)*.

“##” *denotes reference Gupta et al. (2020)*.

“$” *denotes reference Birech et al. (2017)*.

“$$” *denotes reference Perez-Guaita et al. (2016)*.

Specific peaks that exhibit statistically significant changes for a particular elicitor were also observed. Only flg22 treatment in WT showed a statistically significant change in peak intensity at 789.7 cm^−1^. In the literature, this vibrational band region is typically associated with chlorophyll a and phosphodiester bonds (${\text{C}}_{5}^{\prime }$-O-P-O-${\text{C}}_{3}^{\prime }$) in DNA. elf18 treatment specifically resulted in changes for the Raman peak at 1,065 cm^−1^. This band is attributed to skeletal –C–C– stretching of lipids or C–O stretch of cell wall polysaccharides (Movasaghi et al., 2007).

The spread in Raman peak intensities that exhibited changes was further analyzed using histogram plots. It provides a better classification inference with high statistical significance. We found that the Raman shift at 1,521 cm^−1^ and the region between 1,549 and 1,552 cm^−1^ represent carotenoids and proteins (amino acid-tyrosine and tryptophan), respectively, and provide the best discriminating classification between mock and treated plants (Supplementary Figure 7). As the other two carotenoid Raman peaks (1,001 and 1,151 cm^−1^) are present in lower wavenumber regions where there is a significant spectral overlap from other biomolecules such as lipids and proteins, clear differentiation was not observed in these Raman peaks. The changes observed at 1,521 and between 1,549 and 1,552 cm^−1^ are the potential Raman spectral biomarkers, indicating a dynamic early PTI response in plants.

### Raman Spectral Analysis of Pathogen-Infected Plants and Measuring the Infection Response Index

**Figure 6:**
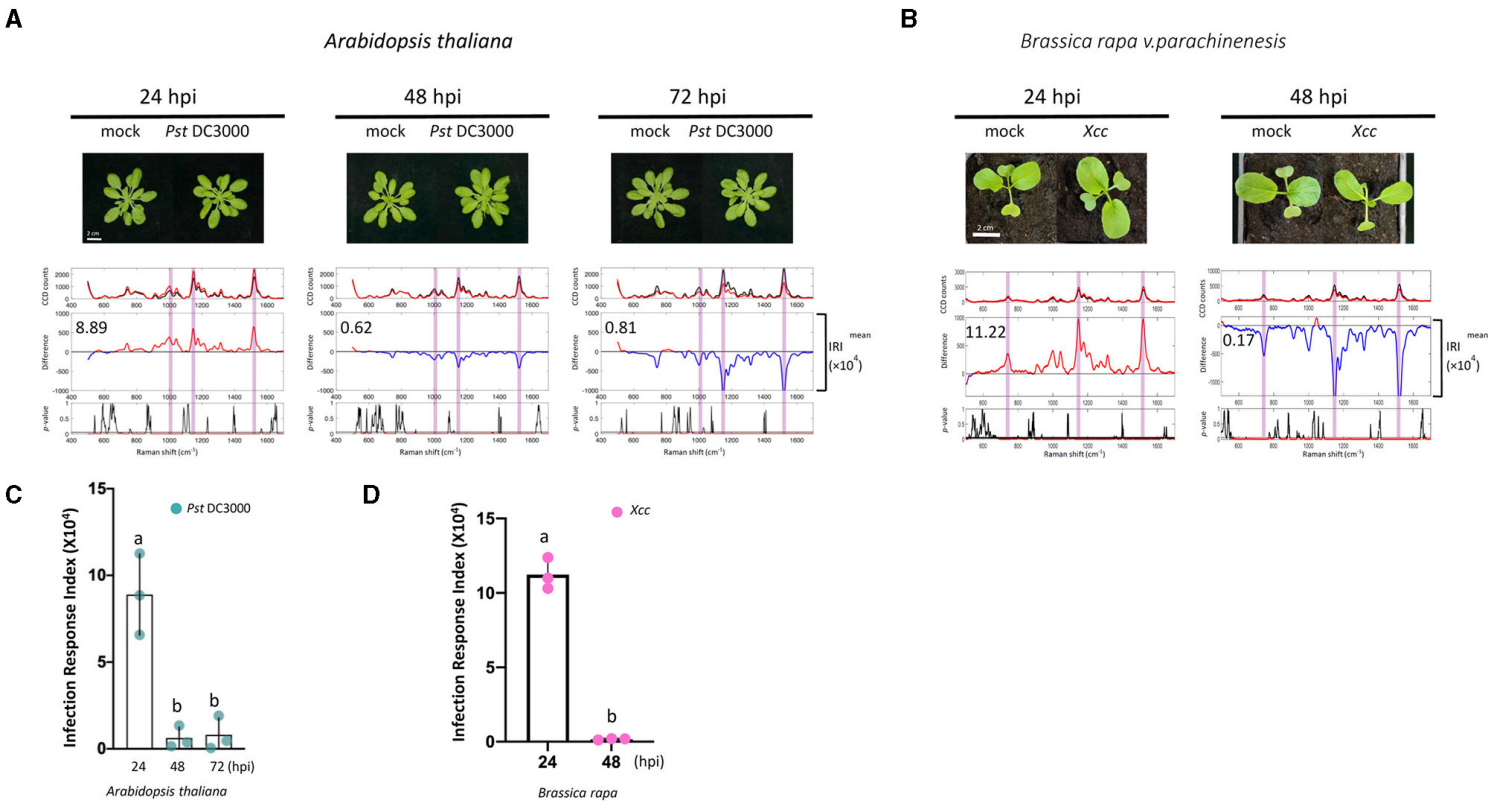
Raman spectra analysis of plants treated with pathogens. **(A,B)** Pathogens [*Pseudomonas syringae pv. Tomato DC3000* **(A)**; *Xanthomonas campestris pv. Campestris* (*Xcc*) **(B)**] were infiltrated with a needleless syringe into Arabidopsis and Choy Sum. Leaf samples taken from the PRs at 24, 48, and 72 hpi were analyzed by Raman spectroscopy. Upper panel: the mean spectra of control (black) and pathogen-treated samples (red). Middle panel: difference spectrum between the mean spectrum of the pathogen-treated sample vs. that of the mock-treated sample. Red indicates that the difference is a positive value, whereas blue indicates a negative value. The number inside the panel indicates the average Infection Response Index (IRI, ×10^4^) value obtained from the three independent experiments. Lower panel: a *t*-test was used to evaluate statistically significant differences between pathogen- and mock-infiltrated samples and the results were expressed as *p*-value. Red indicates *p* < 0.05. **(C,D)** Changes in IRI after the time of infiltration. Left, *P. syringae pv. Tomato DC3000* (green spots), *n* = 3 independent biological samples; Right, *Xcc* (purple spots), *n* = 3 independent biological samples. The results represent the average values of three independent biological experiments with SD. Bars with different letters above are significantly different according to Fisher's least significant difference test (*p* < 0.05).

*Pseudomonas syringae* causes bacterial specks on Arabidopsis and induces chlorosis at later stages of infection (Whalen et al., 1991; Uppalapati et al., 2010; Luo et al., 2017). To see if Raman spectroscopy can be used to detect early Pseudomonas infection, we performed similar experiments in Arabidopsis infected with *Pst* DC3000. In these experiments, we estimated the Infection Response Index (IRI) using the same method as the ERI value. Similar to the elicitor study, the difference Raman spectrum between the infected and mock sample at 24 hpi exhibited high IRI values. The IRI value decreased to almost zero at 48 and 72 hpi (Figures 6A,C).

To further validate the results observed in Arabidopsis in a crop plant, *Xcc* infection in Choy Sum was analyzed. *Xcc* causes Black rot disease, which is a serious problem in all Brassica vegetables resulting in significant yield losses. Typical external visible symptoms of *Xcc* infections are chlorosis and V-shaped lesions in the leaves (Meenu et al., 2013; Nuñez et al., 2018). Under our growth conditions, the characteristic visual symptoms of *Xcc i*nfection are seen within 4–5 days. Raman spectra were measured at 24 and 48 hpi from mock and *Xcc* infiltrated plants to detect spectral changes before the appearance of symptoms. Like Arabidopsis, the difference Raman spectrum between the infected and mock sample at 24 hpi exhibited high IRI values, which decline at 48 hpi (Figures 6B,D). Similar to the Arabidopsis elicitor experiments, callose deposition was also observed in the *Xcc*-infected leaves at 24 hpi (Supplementary Figure 8A).

The vibrational bands contributing toward a high IRI value in Arabidopsis and Choy Sum were largely similar and resembled the ERI spectral data (Table 2; Supplementary Figure 9). Like the elicitor treatment, the most dominant changes upon bacterial infection in Arabidopsis and Choy sum were observed around 1,001, 1,151, and 1,521 cm^−1^, which are attributed to carotenoids (Supplementary Figure 8B). Apart from carotenoids, increase in peak intensities at vibrational bands originating from pectin, phenylpropanoids, cellulose, nitrates, nucleic acids and proteins were the dominant contributors towards the high IRI value. A significant change at 1,253 cm^−1^ peak intensity was seen only in *P. syringae-*infected Arabidopsis, which is associated with proteins and lipids (C–N in-plane stretching), whereas *Xcc-i*nfected Choy sum showed changes at 791.8 cm^−1^ attributed to nucleic acid phosphodiester bond, which were not observed in Arabidopsis infection. As observed with the elicitor study, the Raman shift at 1,521 cm^−1^ and the region between 1,549 and 1,552 cm^−1^ provide the best classification between mock and infected plants.

**Table 2:** Significant vibrational bands and their assignments that contributes to the high IRI value at 24 hrs following *Pseudomonas syringae* and Xcc infection in Arabidopsis and Choy Sum respectively.

**Raman peak (cm^**−1**^)**	**Assignment**	**Molecular bond**
**Arabidopsis**		
742.3	Pectin	γ(C–O–H) of COOH
		ring br. mode (aromatics) [##]
914	Leucine, cellulose, lignin	(C–C), (C–N) stretching [$]
956*	Carotenoids, phosphates	(C–C) [#]
1,001	Phenylalanine, carotenoids	ν3 (C–CH_3_ stretching), phenylalanine ring stretching mode [##]
1,046	Nitrate	NO_3_ stretching [##]
1,151	Carotenoid	C–C stretching; v(C–O–C) [##]
1,180	Carotenoid	dCH, u (pyr half-ring)_as_ [$$]
1,211	Phenylalanine, tryptophan	u (C–C_6_H_5_), u_18_ (dC_m_H) [$$]
1,253*	Proteins, lipids	C–N in plane stretching [#]
1,276	Carotenoid	Amide III [#]
1,318	Cellulose/lignin/protein	dCH2 bending [##]
1,381	Proteins, nucleic acids	dCH3 symmetric [#]
1,433	Proteins, lipids	CH_2_ scissoring [#]
1,521	Carotenoid	–C=C– (in-plane) [#]
1,549	Amide II, proteins: tyrosine, tryptophan, chlorophyll a	Tryptophan, u (C=C) [#]
1,608	Lignin	u (C–C) aromatic ring + s(CH) [##]
**Choy Sum**		
742.8	Pectin	γ(C–O–H) of COOH
		ring br. mode (aromatics) [##]
791.8*	Phosphodiester	(OPO) sym [#]
914	Leucine, cellulose, lignin	(C–C), (C–N) stretching [$]
1,001	Phenylalanine, carotenoids	ν3 (C–CH_3_ stretching), phenylalanine ring stretching mode [##]
1,045	Nitrate	NO_3_ stretching [##]
1,150	Carotenoid	C–C stretching; v(C–O–C) [##]
1,178	Carotenoid	dCH, u (pyr half-ring)_as_ [$$]
1,210	Phenylalanine, tryptophan	u (C–C_6_H_5_), u_18_ (dC_m_H) [$$]
1,277	Carotenoid	Amide III [#]
1,317	Cellulose/lignin/protein	dCH_2_ bending [##]
1,380	Proteins, nucleic acids	dCH_3_ symmetric [#]
1,434	Proteins, lipids	CH_2_ scissoring [#]
1,521	Carotenoid	–C=C– (in-plane) [#]
1,549	Amide II, Proteins: tyrosine, tryptophan, Chlorophyll a	Tryptophan, u (C=C) [#]
1,608	Lignin	u (C–C) aromatic ring + s(CH) [##]

“*” *denotes vibrational bands, which were specific to each pathogen*.

“#” *denotes reference Movasaghi et al. (2007)*.

“##” *denotes reference Gupta et al. (2020)*.

“$” *denotes reference Birech et al. (2017)*.

“$$” *denotes reference Perez-Guaita et al. (2016)*.

## Discussion

In this study, we have provided evidence that the infiltration of WT Arabidopsis leaves with an elicitor result in reproducible changes in the Raman spectra acquired from the leaf region proximal to the site of elicitor infiltration, indicating that the Raman spectral changes are associated with PTI response (Figures 1, 2).

The difference Raman spectrum between the elicitor-treated and mock-treated leaf shows clear and reproducible changes in the Raman shift from about 800 to 1,600 cm^−1^. These changes are remarkably similar in plants of various genotypes when a positive PTI is expected. Using statistical methods and employing a simple mathematical formula, we arrived at the ERI, which provides a quantitative measure of the degree of PTI as determined by the difference Raman spectrum (Supplementary Figure 2). Our results showed that upon the treatment with 1 μM flg22 the ERI value at 10 hpi was 1.12 × 10^4^, which increased about 8 times by 24 hpi. Similar results were obtained using elf18 (Figures 3, 4).

The physiological significance of the difference Raman spectrum and the ERI values obtained with WT plants is further strengthened by analysis with several Arabidopsis mutants that are deficient in PRRs and downstream signaling components. The mutants that are defective in the positive regulation of PTI response show low ERI values (< 1 × 10^4^), whereas the mutants that are hyperresponsive to elicitor treatment display high ERI values at an earlier time point. This indicates that the ERI values can be used as a potential measurement of an active and robust PTI response in plants.

An examination of the difference Raman spectrum obtained from elicitor/pathogen studies uncovered a significant increase in peak intensity in spectral regions as denoted in Tables 1, 2. The changes in Raman peaks contributing toward the high ERI or high IRI value at 24 h with both the elicitor and pathogens are largely identical, indicating that metabolome changes triggered by elicitors and pathogen are similar. A difference in the magnitude of the IRI factor between Arabidopsis and Choy Sum can be expected because different pathogens can trigger varied immune responses.

The most significant increase in peak intensity under all types of elicitor/pathogen treatment was observed at 1,001, 1,151, and 1,521 cm^−1^, which correspond to the characteristic bands attributed to carotenoids (Baranski et al., 2005; Schulz et al., 2005; Lorenz et al., 2017). These results suggest that there is a transient increase in the carotenoid upon elicitor treatment or infection. A transient carotenoid increase as an early event of PTI has not been previously reported, and its physiological significance remains unclear at this point. Recent research has shown that carotenoids are constantly being synthesized and also degraded to produce other derivatives (Beisel et al., 2010). Apocarotenoids are produced by oxidative cleavage of carotenoids either catalyzed by carotenoid cleavage dioxygenases (CCDs) or by non-enzymatic processes. New studies are unraveling the functions of apocarotenoids in plant development and stress response. Important plant apocarotenoids include phytohormones such as abscisic acid (ABA) and strigolactones and signaling metabolites like β-cyclocitral (Felemban et al., 2019). In plants, phytohormone ABA (Supplementary Figure 6) plays a role in abiotic/biotic stress response (Jia et al., 2017). A gene analysis showed that the expression levels of *NCED*, which encodes the rate-limiting enzyme for ABA biosynthesis, are highly elevated 3–6 hpi by either elicitor. The expression levels of various CCD genes are also found to be altered by elicitor treatment (Supplementary Figure 6). This result is consistent with the notion that the transiently increased carotenoids are subsequently fragmented and modified to produce ABA and other derivatives.

In addition to carotenoids, Raman spectral changes implicate a transient increase in metabolites like pectin, phenylpropanoids, cellulose, nitrates, lignin, nucleic acid, and proteins to be involved in early PTI response. A previous study, which evaluated metabolic changes associated with *P. syringae* infection in Arabidopsis at different time points, observed the changes as early as 8 hpi. The major metabolic pathways perturbed upon infection were phenylpropanoid/lignins, tryptophan/indolic glucosinolates/nicotinic acid, methionine/aliphatic glucosinolates, purine/riboflavin/folate, and urea cycle/polyamine/proline metabolic pathways. Other pathways found to be affected by infection include carotenoid metabolism, chlorophyll degradation, and nonaromatic amino acid metabolism (Ward et al., 2010). The Raman spectral analysis also indicates changes in similar metabolites upon infection in Arabidopsis. Along with carotenoids, the changes between 1,549 and 1,552 cm^−1^ regions were found to discriminate between the mock and infected plants with high statistical significance. This vibration band is associated with Amide II (C=N and N–H stretch): mainly proteins, tyrosine, and tryptophan (Movasaghi et al., 2007). Ward et al. (2010) in their study found that amino acids, tryptophan, and tyrosine are significantly increased in abundance by 12 hpi with *P. syringae* in Arabidopsis. Our study also identified the genes involved in tryptophan metabolism altered by elicitor treatment (Supplementary Figure 1). The Raman shift was observed at 1,549 and 1,552 cm^−1^ under elicitation, and infection probably reflects an increase in the abundance of tryptophan and tyrosine.

Several groups have used Raman spectroscopy to analyze a number of crop plants infected with bacterial, fungal, and viral pathogens, and, in most cases, a decrease in carotenoid was consistently seen; however, a decrease in carotenoid has also been observed with abiotic stresses. We too have previously detected a decrease in carotenoid contents in the leaf blades and petioles of Arabidopsis with shade avoidance syndrome (SAS) using Raman spectroscopy (Altangerel et al., 2017; Sng et al., 2020). By contrast, we found that a transient increase in carotenoids is an early event in PTI. We note that the carotenoid increase detected in our study occurs at about 10–24 h upon receptor activation before the manifestation of any disease symptoms. By contrast, in most of the previous works, the Raman spectra were taken with plants likely to be several days after pathogen infection as the tissues were already showing disease symptoms.

Plant diseases are known to be a major factor compromising crop yield worldwide (Velásquez et al., 2018; Savary et al., 2019), and the early detection of pathogen infection can greatly facilitate disease management. The use of ERI as a diagnostic Raman signature for early PTI will provide an important tool for the identification of plants with early pathogen infection and for facilitating effective disease management. The availability of a high-throughput custom-made portable or hand-held Raman spectrometer (Farber and Kurouski, 2018; Krimmer et al., 2019; Sanchez et al., 2019) would allow the Raman spectral analysis to be performed directly with field-grown crops in a noninvasive manner, and our group is currently developing such systems (Gupta et al., 2020).

## Data Availability Statement

The raw data supporting the conclusions of this article will be made available by the authors, without undue reservation.

## Author Contributions

PC, GS, JS, RR, RS, and N-HC designed the experiments. PC, H-ZM, PD, GS, C-HH, and SK executed the experiments. PC and GS generated scripts for analysis using the MATLAB software. All authors participated in the interpretation and discussion of results. PC, GS, RR, RS, and N-HC wrote the manuscript. All authors contributed to the article and approved the submitted version.

## Funding

This research was supported by the National Research Foundation (NRF), Primer Minister's Office, Singapore under its Campus for Research Excellence and Technological Enterprise (CREATE) program. The Disruptive and Sustainable Technology for Agricultural Precision (DiSTAP) is an Interdisciplinary Research Group (IRG) of the Singapore MIT Alliance for Research and Technology Centre (SMART).

## Conflict of Interest

The authors declare that the research was conducted in the absence of any commercial or financial relationships that could be construed as a potential conflict of interest.

## Publisher's Note

All claims expressed in this article are solely those of the authors and do not necessarily represent those of their affiliated organizations, or those of the publisher, the editors and the reviewers. Any product that may be evaluated in this article, or claim that may be made by its manufacturer, is not guaranteed or endorsed by the publisher.
