# Supplementary material for: Rapid Detection and Quantification of Plant Innate Immunity Response Using Raman Spectroscopy
Source: Front Plant Sci. 2021 Oct 21;12:746586. doi: 10.3389/fpls.2021.746586 (PMC8566886; doi:10.3389/fpls.2021.746586)

**Supplementary figures**

**
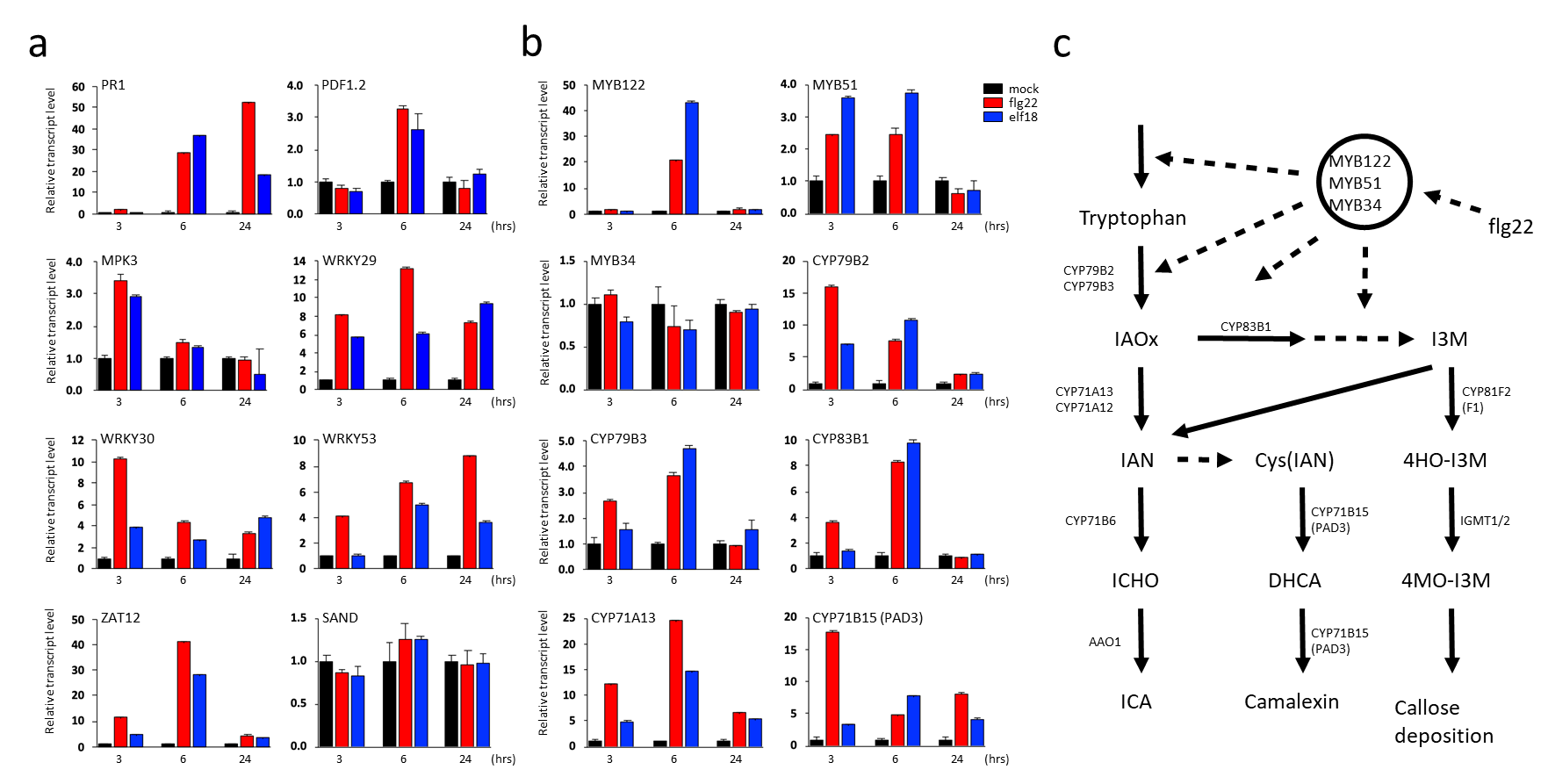
**

**Fig. S1.** Induction of immune response genes by elicitors. Leaves were collected at the indicated time points after infiltration with 1 µM elicitor (flg22, red bar; elf18, blue bar) and transcript levels were determined by RT-qPCR. Transcript levels of *Act2* (*Actin2*, At3g18780) were used for normalization. Expression values of elicitor treated samples were shown relative to the levels obtained with mock treatment (black bar) and the average values of 3 technical replicates were shown with standard deviation (s.d.). Similar results were obtained from three independent biological experiments. (a) Induction of expression of immune-response related genes after flg22 and elf18 treatment. Abbreviation: *WRKY53* (At4g23810), early senescence WRKY networking regulator; *WRKY29* (At4g23550); *WRKY30* (At5g24110); *ZAT12* (At5g59820), ZINC FINGER OF ARABIDOPSIS THALIANA12; *PR1* (At2g14610), PATHOGENESIS-RELATED GENE1); *PDF1.2* (At5g44420), PLANT DEFENSIN; *MPK3* (At3g45640), Mitogen-activated Protein Kinase3; *SAND* (At2g28390), MONENSIN SENSITIVITY1 (MON1), SAND family protein. *SAND* was used as a control as its expression was not affected by elicitor infiltration. , time post infiltration (hrs). (b) Effect of flg22 and elf18 treatment on the expression of tryptophan-metabolism related genes. *MYB12*2 (At1g74080), *MYB51* (At1g18570), *MYB34* (At5g60890), *CYP79B2* (At4g39950), *CYP79B3* (At2g22330), *CYP83B1* (At4g31500), *CYP71A13* (At2g30770), *CYP71B15*/*pad3* (At3g26830). (c) A diagram showing tryptophan metabolism pathways and related structural and regulatory genes. Treatment of flg22 induces *MYB122*, *MYB51*, and *MYB34* (Frerigmann *et al*., 2016).


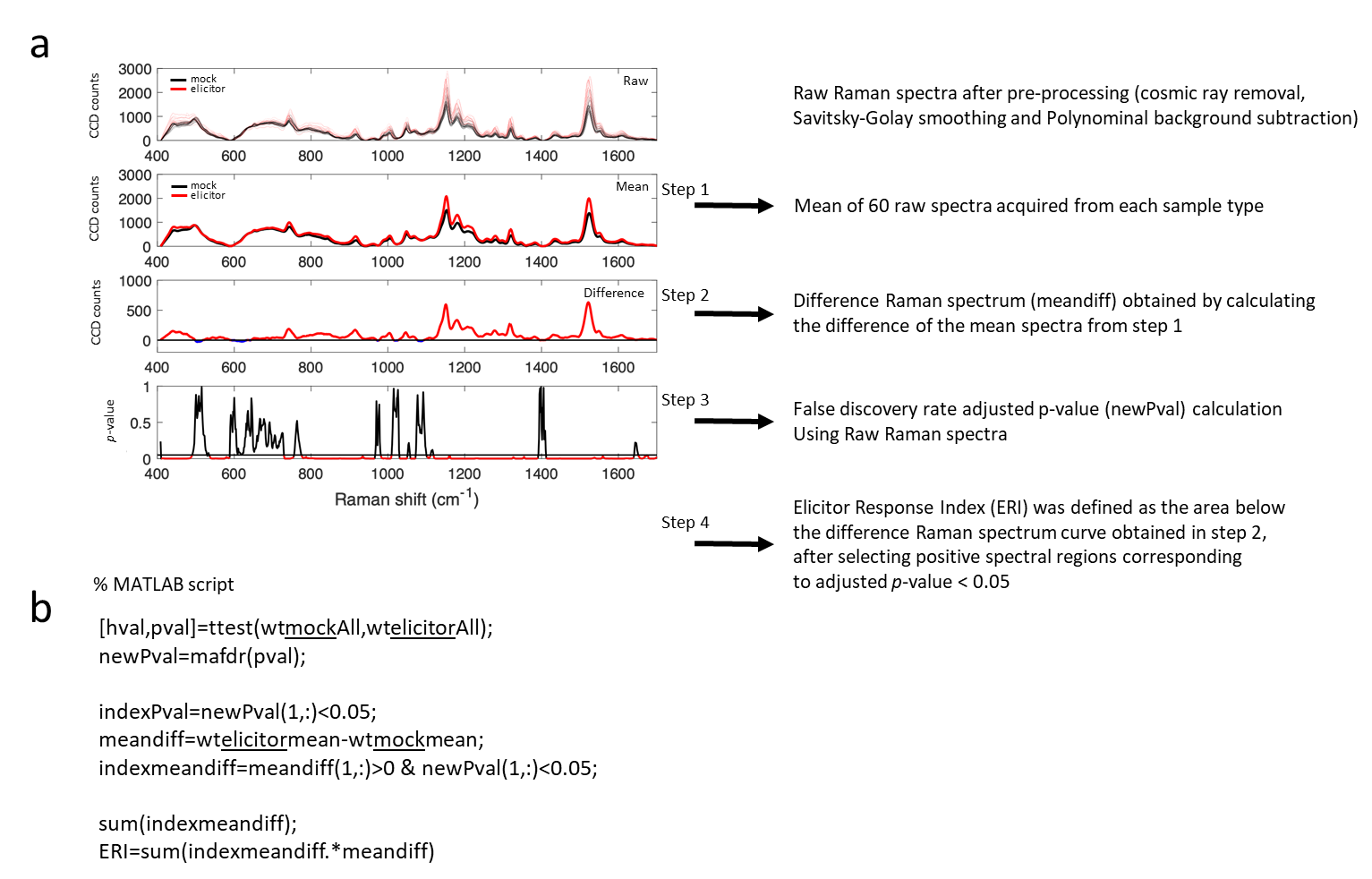


**Fig. S2.** Raman spectral analysis and calculation of the elicitor response index (ERI). (a) Top panel: Raw Raman spectra obtained from mock control and elicitor-treated samples after spectral pre-processing (cosmic ray removal, Savitsky-Golay smoothing, and polynomial background subtraction). Second panel: Mean Raman spectra obtained from control and elicitor-treated samples (Mean plots). Third panel: Difference Raman spectrum of elicitor-treated versus control samples (Difference of Mean plot). Bottom panel: False discovery rate adjusted *p*-value calculation by performing t-test on Raw Raman spectra. ERI was defined as the area below the Difference Raman spectrum curve, shown above in the third panel, after selecting positive spectral regions corresponding to adjusted *p*-value < 0.05 (b) MATLAB programming language script used to calculate ERI.


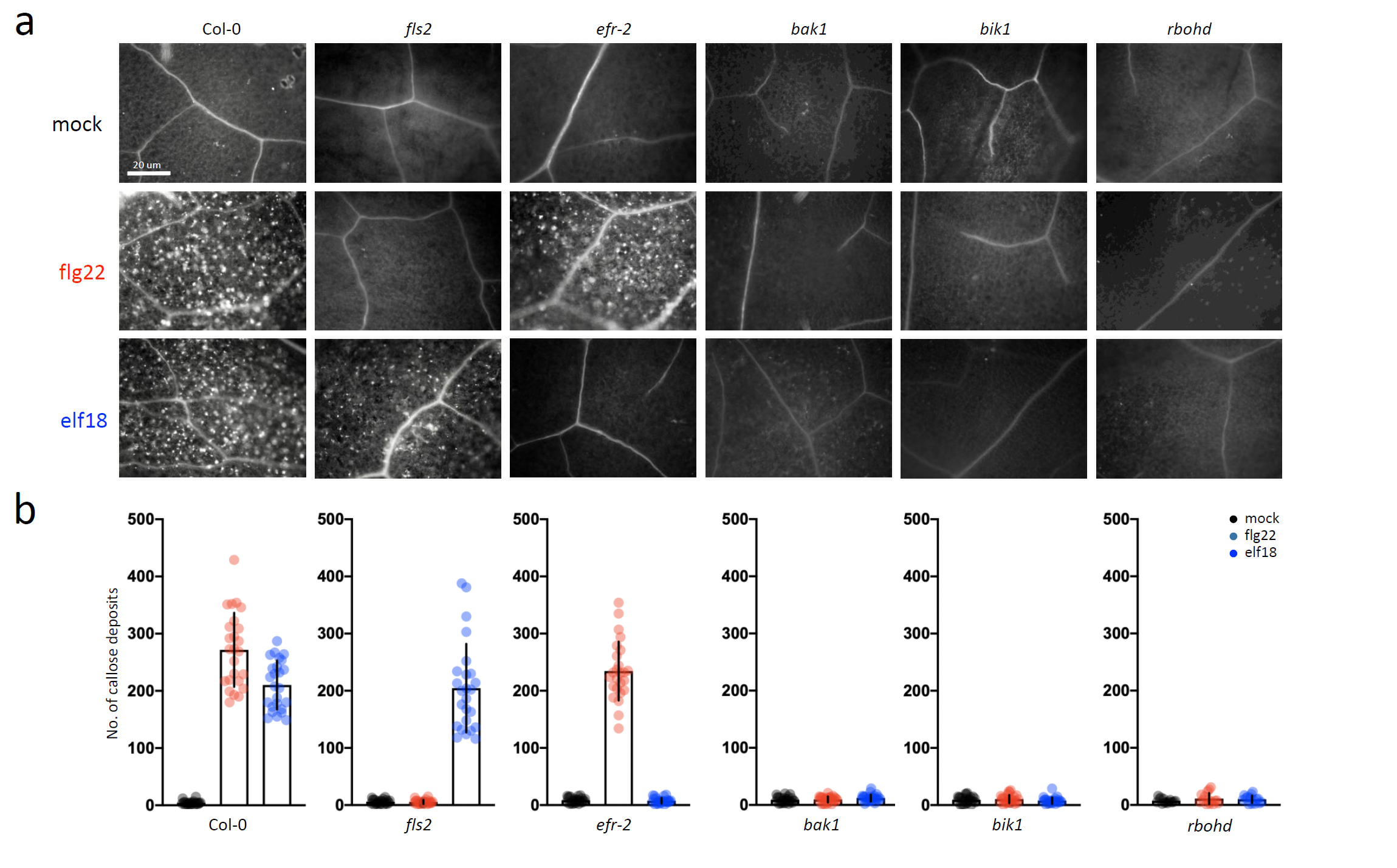


**Fig. S3.** Determination of callose deposition in WT and mutant plants after elicitor treatment. Callose deposition in the rosette leaves of 5-week-old Arabidopsis WT (Col-0), PAMP-associated mutants (*fls2*, *efr-2*, *bak1*, *bik1*, *rbohd*), and wounding-related mutant (*glr3.3glr3.6*) treated for 24 hr with H_2_O, or 1 µM flg22, or 1 µM elf18. (a) Representative pictures of aniline-blue stained callose fluorescence; Scale bars = 20 um. (b) Quantification of callose deposition by aniline blue-staining at 24 hr after infiltration of mock (grey bar), 1 µM flg22 (red bar), and 1 µM elf18 (blue bar). Values shown are mean ± s.d. of 25-30 surveyed leaf regions. Each region covered an area of 5,996 µm^2^. For each experiment, the number of callose was measured in 2 infiltrated plants with about 4-5 leaf regions surveyed per plant. This gave a total of about 8-10 data points per experiment. The experiment was repeated with 3 independent biological samples. The graph shows average value with n=25-30.

**
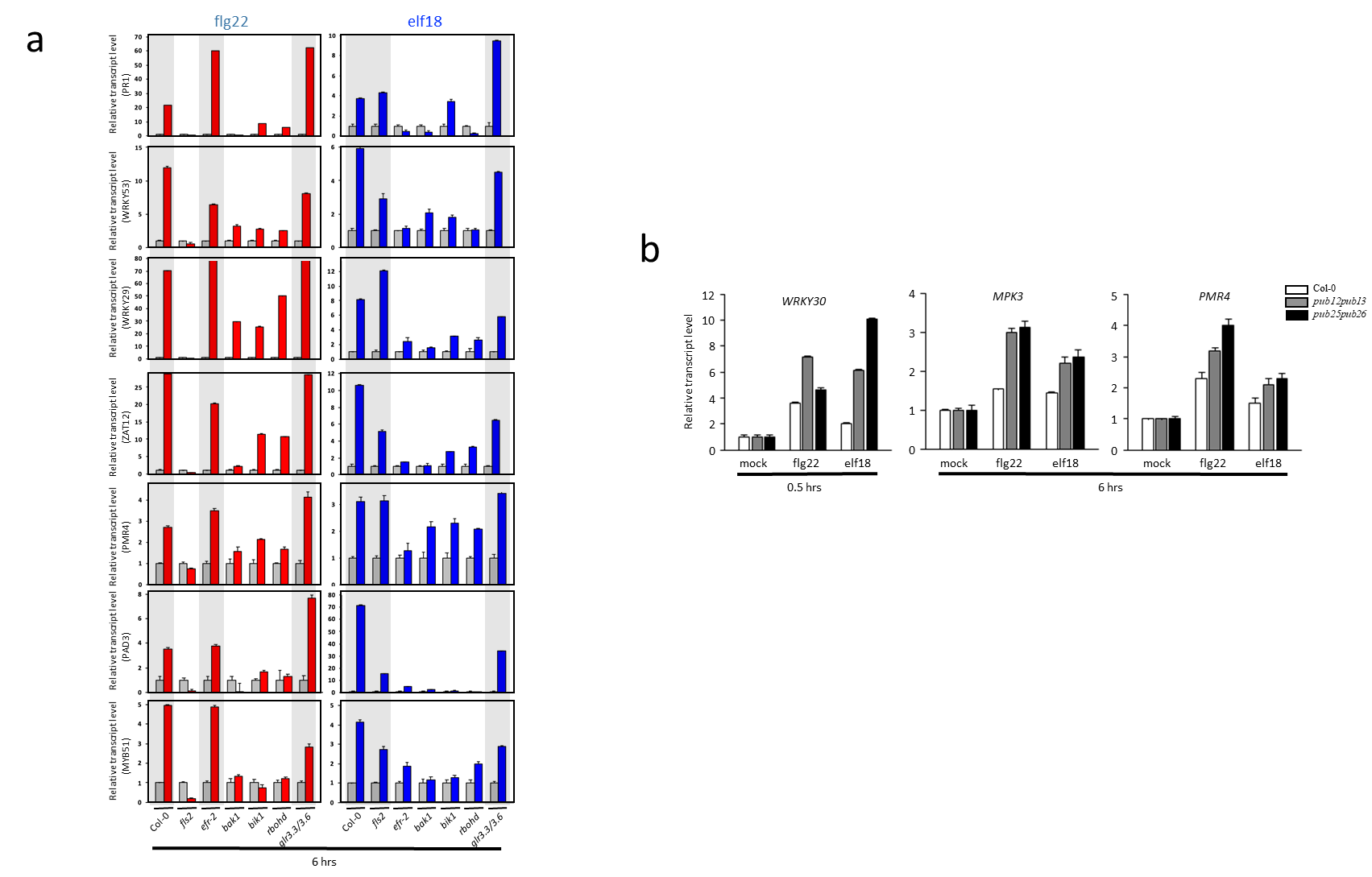
**

**Fig. S4.** Induction of PTI marker genes in response to elicitor treatment (flg22 and elf18) in various mutants. (a) 5-week-old plants of *Arabidopsis thaliana* ecotype Columbia (Col-0), *fls2* (salk_062054), *efr-2* (salk_068675), *bak1* (salk_116202), *bik1* (salk_005291), *rbohd* (salk_083046), and *glr3.3/3.6* loss-of-function mutants were treated with flg22 or elf18, at a final concentration of 1 µM, and leaf samples collected at 6 hrs (time post infiltration) to analyze the expression of *PR1* (At2g14610), pathogenesis-related gene 1; *WRKY53* (At4g23810), *WRKY29* (At4g23550), *WRKY30* (At5g24110), *ZAT12* (At5g59820); a zinc finger protein, *PMR4* (At4g03550), *PAD3* (At3g26830), *WRKY30* (At5g24110) and *MYB51* (At1g18570) as PTI marker genes by RT-qPCR. (b) 5-week-old plants of *Arabidopsis thaliana* ecotype Columbia (Col-0), *pub12/13* and pub25/26 loss-of-function mutants were treated with flg22 or elf18, at a final concentration of 1 µM, and leaf samples collected at 0.5 and 6 hrs (time post infiltration) to analyze the expression of *WRKY30* (At5g24110), *WRKY30* (At5g24110), *PMR4* (At4g03550) genes by RT-qPCR. The relative fold change ± s.d. of the PAMP-responsive genes was obtained from a comparison between the PAMP-treated plants and mock-treated plants with the reference gene *act1* (At3g18780). The average values of 3 technical replicates were shown with s.d., and the similar results were obtained from three independent biological experiments.


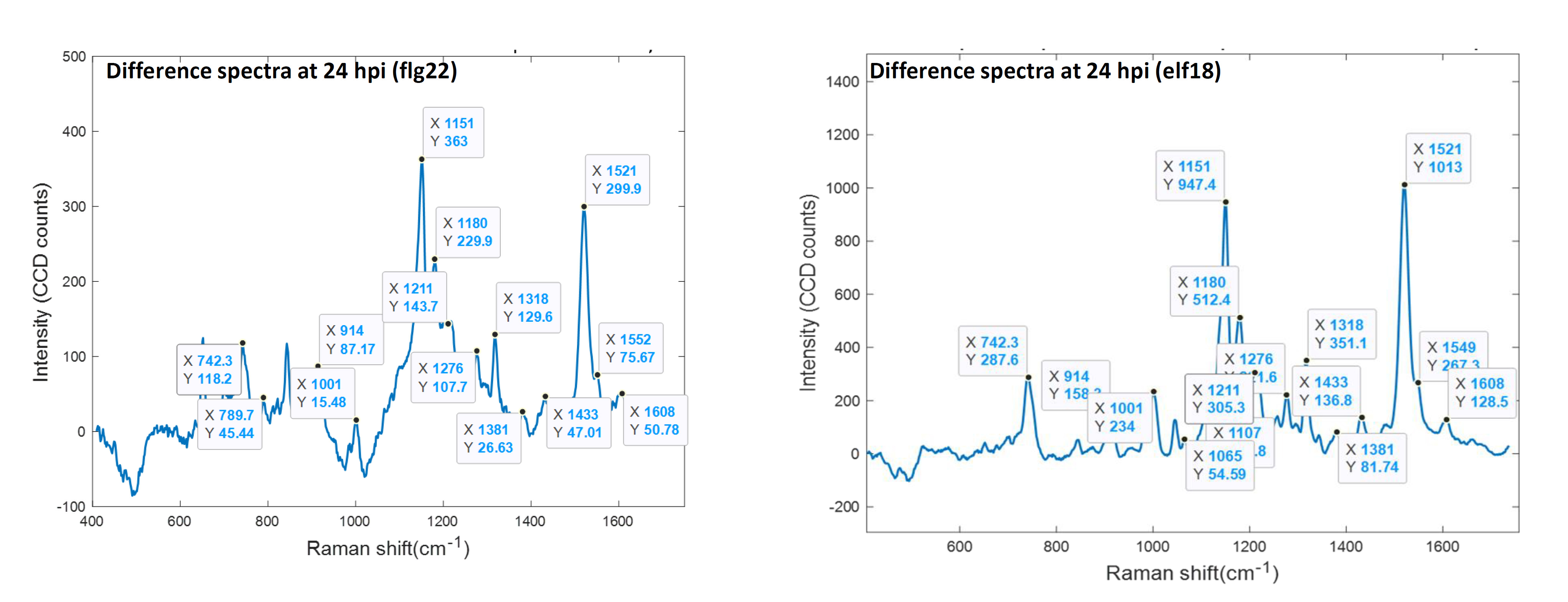


**Fig. S5.** Difference Raman spectrum of elicitor-treated versus control samples and Raman peak assignment


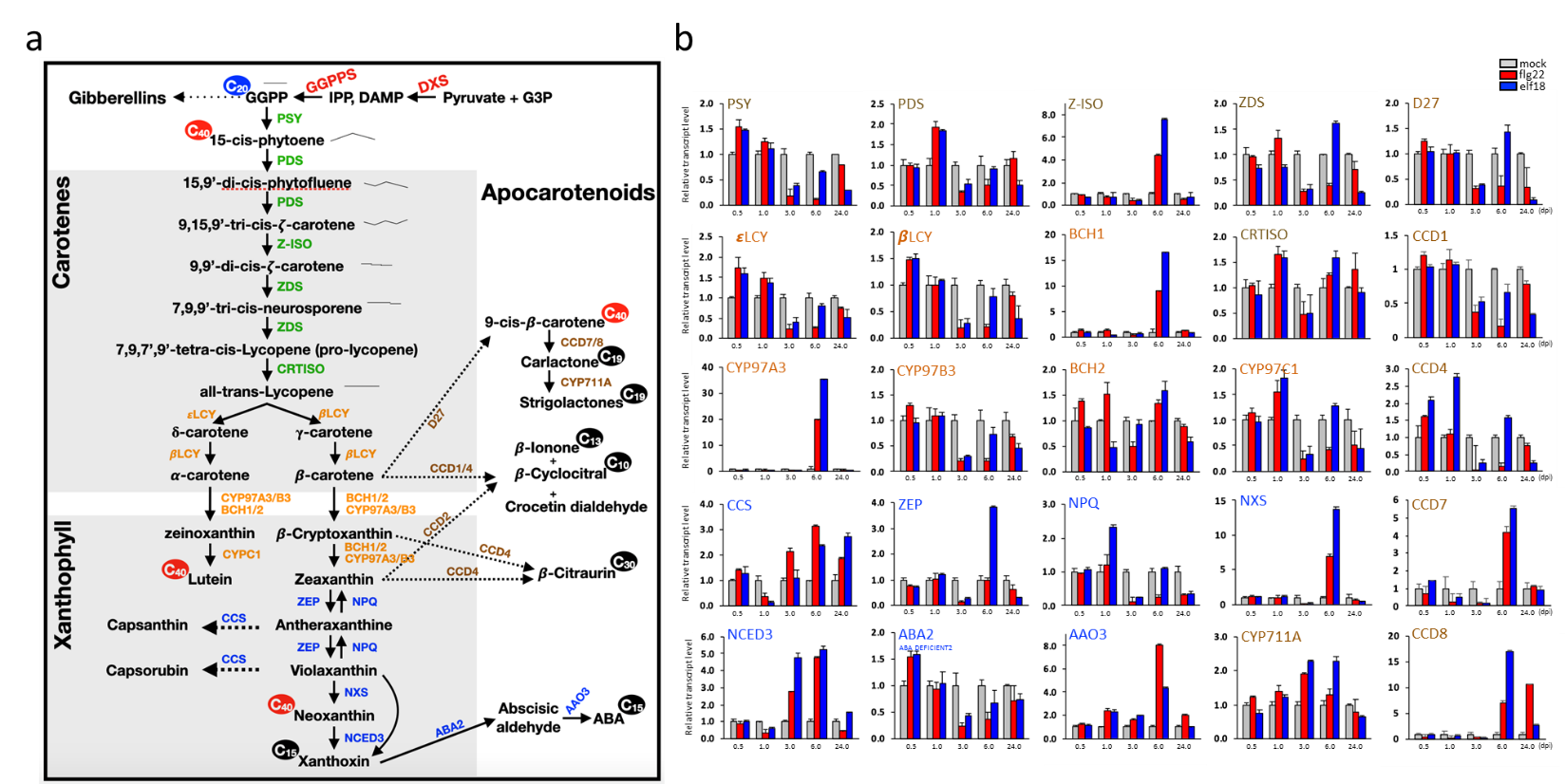


**Fig. S6.** The general carotenoid metabolic pathway in Arabidopsis and expression changes of carotenoid biosynthetic genes after elicitor treatment. (a) A schematic diagram showing carotenoids biosynthesis and cleavage pathways in plants. (b) Expression levels of carotenoids biosynthetic genes at various times post infiltration (hrs) after elicitor treatment. Enzymes (in color) and corresponding Arabidopsis mutant names (in italics) are listed. *PSY*, phytoene synthase (At5g17230); *PDS*, phytoene desaturase (At4g14210); *Z-ISO*, z-carotene isomerase (At1g10830); *ZDS*, z-carotene desaturase (At3g04870); *CRTISO*, carotenoid isomerase (At1g06820); *εLCY*, Lycopene ε-cyclase (LUT2, At5g57030); *βLCY*, Lycopene β-cyclase (At3g10230); *CYP97A3*, (LUT5, At1g31800); *CYP97C1*, (LUT1, At3g53130); *CYP97B3*, (At4g15110); *BCH1*, β-carotene hydroxylase 1 (At4g25700); *BCH2*, β-carotene hydroxylase 2 (At5g52570); *CCS*, COPPER CHAPERONE FOR SOD1(At1g12520); *ZEP*, Zeaxanthin epoxidase (ABA1, At5g67030); *NPQ*, NON-PHOTOCHEMICAL QUENCHING1 (At1g08550), *NXS*, neoxanthin synthase (ABA4, At1g67080); *NCED3*, 9-cis-epoxycarotenoid dioxygenase (At3g14440); *ABA2*, ABA DEFICIENT 2 (At1g52340); *AAO3*, ABSCISIC ALDEHYDE OXIDASE3 (At2g27150); *CCD1*, carotenoid cleavage dioxygenase1 (At3g63520); *CCD4*, At4g19170; *CCD7*, At2g44990; *CCD8*, At4g32810; *D27*, β-carotene isomerase (At1g64680); *CYP711A*, (At2g26170). Transcript levels of *Act2* (Actin2, At3g18780) were used for normalization. Expression values of elicitor treated samples were shown relative to the levels obtained with mock treatment (grey bar) or 1 µM elicitor (flg22, red bar; elf18, blue bar) and the average values of 3 technical replicates were shown with s.d. The similar results were obtained from two independent biological experiments.


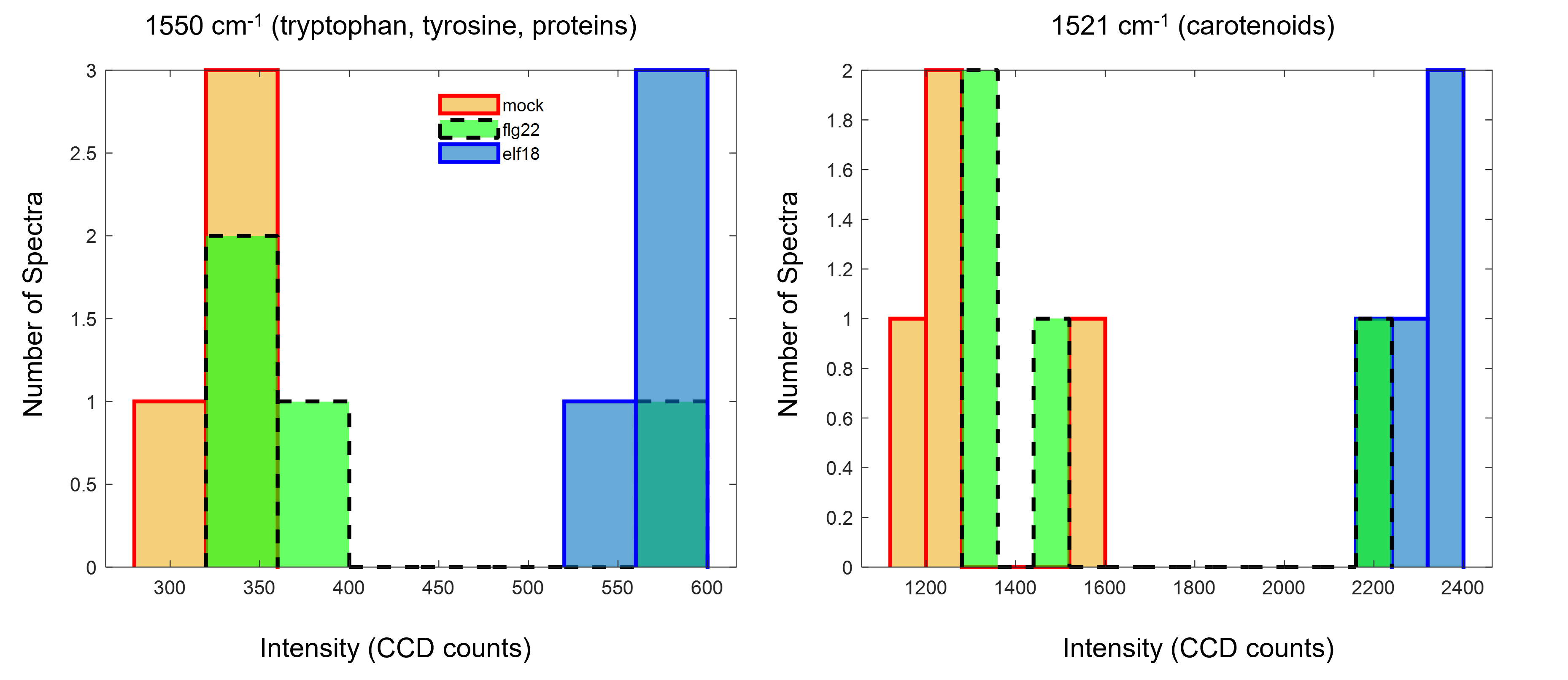


**Fig. S7** Spread in Raman peak intensities analysed by histogram plots: Besides the main carotenoid Raman peak at wavenumber 1521 cm^-1^, it was observed that Raman shift wavenumber at 1550 cm^-1^ could also clearly distinguish between mock and elicitor treated samples. Histogram analysis was performed to make sure that data was not skewed by an outlier. All Raman shift wavenumbers mentioned in the Table 1 were analysed using his histogram analysis.


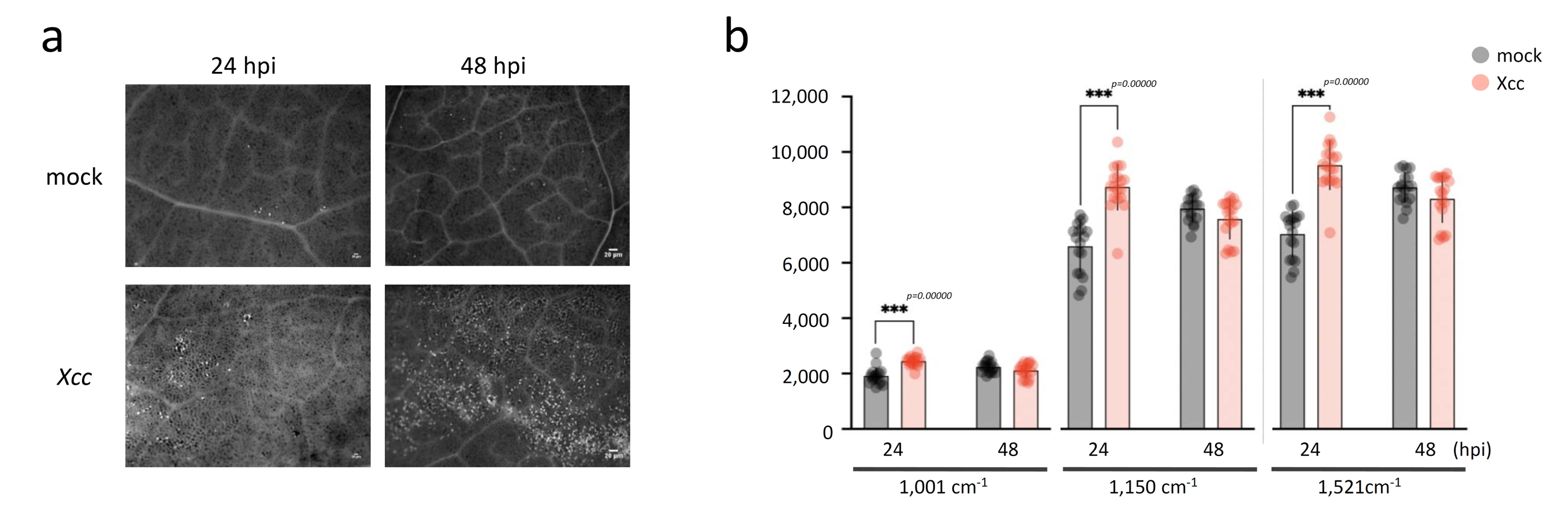


**Fig. S8.** Callose deposition and carotenoid peak intensity changes in Choy Sum leaf samples treated with *Xcc* after 24 and 48 hpi **a)** Representative images of aniline-blue stained callose deposition in the mock and *Xcc* infiltrated Choy Sum leaves.; Scale bars = 20 µm.

(b) Raman shifts at 1,001 cm^-1^, 1,151 cm^-1^ and 1,521 cm^-1^ are attributed to carotenoids. n=3 independent biological samples.


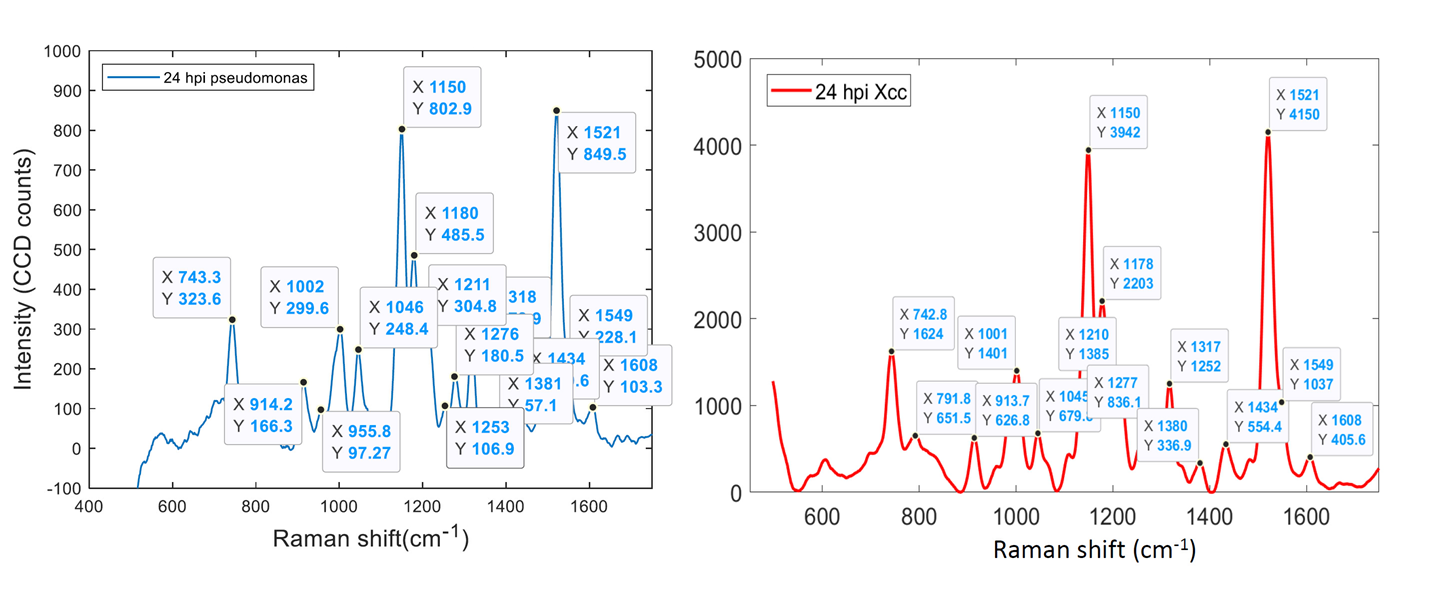


**Fig. S9.** Difference Raman spectrum of pathogen infected versus control samples showing the Raman peak assignment

**Table S1** Primer sets for RT-qPCR


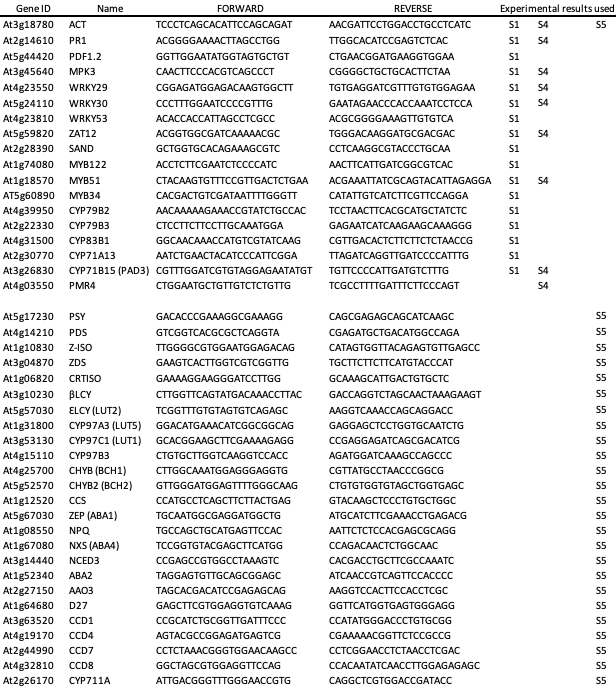

Supplement: Supplementary file 1 [file Data_Sheet_1.docx]
